# Epigenome-Guided Analysis of the Transcriptome of Plaque Macrophages during Atherosclerosis Regression Reveals Activation of the Wnt Signaling Pathway

**DOI:** 10.1371/journal.pgen.1004828

**Published:** 2014-12-04

**Authors:** Stephen A. Ramsey, Yuliya Vengrenyuk, Prashanthi Menon, Irina Podolsky, Jonathan E. Feig, Alan Aderem, Edward A. Fisher, Elizabeth S. Gold

**Affiliations:** 1 Department of Biomedical Sciences and School of Electrical Engineering and Computer Science, Oregon State University, Corvallis, Oregon, United States of America; 2 Division of Cardiology, School of Medicine, New York University, New York, New York, United States of America; 3 Seattle Biomedical Research Institute, Seattle, Washington, United States of America; University of Pennsylvania, United States of America

## Abstract

We report the first systems biology investigation of regulators controlling arterial plaque macrophage transcriptional changes in response to lipid lowering *in vivo* in two distinct mouse models of atherosclerosis regression. Transcriptome measurements from plaque macrophages from the *Reversa* mouse were integrated with measurements from an aortic transplant-based mouse model of plaque regression. Functional relevance of the genes detected as differentially expressed in plaque macrophages in response to lipid lowering *in vivo* was assessed through analysis of gene functional annotations, overlap with *in vitro* foam cell studies, and overlap of associated eQTLs with human atherosclerosis/CAD risk SNPs. To identify transcription factors that control plaque macrophage responses to lipid lowering *in vivo*, we used an integrative strategy – leveraging macrophage epigenomic measurements – to detect enrichment of transcription factor binding sites upstream of genes that are differentially expressed in plaque macrophages during regression. The integrated analysis uncovered eight transcription factor binding site elements that were statistically overrepresented within the 5′ regulatory regions of genes that were upregulated in plaque macrophages in the *Reversa* model under maximal regression conditions and within the 5′ regulatory regions of genes that were upregulated in the aortic transplant model during regression. Of these, the TCF/LEF binding site was present in promoters of upregulated genes related to cell motility, suggesting that the canonical Wnt signaling pathway may be activated in plaque macrophages during regression. We validated this network-based prediction by demonstrating that β-catenin expression is higher in regressing (vs. control group) plaques in both regression models, and we further demonstrated that stimulation of canonical Wnt signaling increases macrophage migration *in vitro*. These results suggest involvement of canonical Wnt signaling in macrophage emigration from the plaque during lipid lowering-induced regression, and they illustrate the discovery potential of an epigenome-guided, systems approach to understanding atherosclerosis regression.

## Author Summary

Atherosclerosis, a progressive accumulation of lipid-rich plaque within arteries, is an inflammatory disease in which the response of macrophages (a key cell type of the innate immune system) to plasma lipoproteins plays a central role. In humans, the goal of significantly reducing already-established plaque through drug treatments, including statins, remains elusive. In mice, atherosclerosis can be reversed by experimental manipulations that lower circulating lipid levels. A common feature of many regression models is that macrophages transition to a less inflammatory state and emigrate from the plaque. While the molecular regulators that control these responses are largely unknown, we hypothesized that by integrating global measurements of macrophage gene expression in regressing plaques with measurements of the macrophage chromatin landscape, we could identify key molecules that control macrophage responses to the lowering of circulating lipid levels. Our systems biology analysis of plaque macrophages yielded a network in which the Wnt signaling pathway emerged as a candidate upstream regulator. Wnt signaling is known to affect both inflammation and the ability of macrophages to migrate from one location to another, and our targeted validation studies provide evidence that Wnt signaling is increased in plaque macrophages during regression. Our findings both demonstrate the power of a systems approach to uncover candidate regulators of regression and to identify a potential new therapeutic target.

## Introduction

Underlying many cardiovascular diseases is atherosclerosis, an arterial accumulation of lipid-laden macrophages (foam cells) with attendant chronic inflammation [1]. Atherosclerosis is endemic in adults [2] and can progress over decades before presenting with overt symptoms [3]. Atherosclerosis is correlated with risk factors such as hyperlipidemia, especially high plasma levels of low-density lipoprotein (LDL) cholesterol [4], [5]. There is significant interest in finding new therapies to promote the regression of atherosclerotic plaques [6]–[8], with particular emphasis on the plaque macrophage as a potential target because of its roles in lipoprotein uptake and vascular inflammation [9]–[12], plaque destabilization [13], [14], and plaque remodeling in response to dietary changes [15].

In mice, plaque regression has been achieved through expression [16]–[18] or inactivation [19], [20] of lipid metabolism-modifying genes; aortic tissue grafting [21]–[23]; and treatment with small-molecule therapeutics [24]–[26] or biologics [27], [28], frequently in conjunction with a diet alteration. We have been studying the role of macrophages during plaque regression in two mouse models of regression, the *Reversa* mouse [19], [20], [29], [30] and an aortic arch transplant model [21], [22], [31]. *Reversa* mice are *Ldlr* ^−/−^ *Apob* ^100/100^ [32], [33] and they possess a loxP-flanked allele of the gene encoding microsomal triglyceride transfer protein (*Mttp*) and a drug-inducible Cre recombinase (*Cre*) transgene [19], [34]. In the *Reversa* mouse, hyperlipidemia can be reversed by the transient induction of *Cre* expression in the liver, which causes recombinant knockout of *Mttp*, and thus significantly lowers the plasma levels of very low density lipoprotein (VLDL) and LDL [19]. In prior work we have shown that inactivation of *Mttp* in the *Reversa* mouse, when combined with a switch from Western diet to chow, leads to plaque regression at the aortic root [20]. In the aortic transplant model, an atherosclerotic aortic arch from an *Apoe* ^−/−^ mouse [35] is grafted in place of a segment of the abdominal aorta [31] of either a wild-type (WT) or an *Apoe* ^−/−^ mouse. Grafts within WT recipients exhibit plaque regression, while plaques within *Apoe* ^−/^ recipients continue to progress [22]. A common feature of the *Reversa* and transplant regression models is the substantial reduction in plaque macrophages during regression [20], [23], [29], [30], [36]. It is at present unknown whether there are molecular pathways that may initiate the depletion of plaque macrophages that are shared across regression models. Identifying such core pathways could yield new therapeutic approaches to stimulate regression and/or beneficial remodeling of plaque.

Recently, we studied transcriptome differences between macrophages in regressing versus progressing plaques in the aortic transplant model [36]. We observed patterns of differential expression that suggest that macrophages in regressing plaques up-regulate genes associated with cell motility, down-regulate genes associated with cell adhesion, and up-regulate genes associated with an anti-inflammatory, “M2 macrophage” [37] phenotype. Although our analysis pointed to several candidate molecular regulators, it is unknown whether there are transcription factors that are common to the transplant and *Reversa* regression models that act as master controllers for the responses of plaque macrophages to lipid lowering.

In the current study we carried out a systems investigation of the *Reversa* model, with three goals: (i) to identify pathways and gene functions that are associated with plaque macrophage responses to lipid lowering *in vivo*; (ii) to understand the connections between gene sets identified in our study and gene sets from previous studies of macrophage foam cell formation, aortic wall changes during plaque progression and regression, and atherosclerosis risk alleles in humans; and (iii) to identify transcriptional regulators that are associated with macrophage responses to lipid lowering *in vivo*. We therefore carried out microarray transcriptome profiling of plaque macrophages isolated from the aortic root of *Reversa* mice undergoing lipid lowering *in vivo* and analyzed differentially expressed genes using multiple bioinformatics approaches. Gene functional enrichment analysis (i) detected over-representation of cytoskeletal-binding and Rho GTPase genes among genes that are upregulated in response to lipid lowering, pointing to cytoskeletal reorganization during regression. Comparative analysis (ii) of gene sets from our study with those from previous studies of *in vitro* models of macrophage foam cell formation revealed a statistically significant overlap. Additionally, we found a significant overlap between human monocyte expression quantitative trait loci (eQTLs) for human orthologs of genes that are differentially expressed during plaque regression, and genetic loci that are associated with risk of atherosclerosis or coronary artery disease. Promoters of genes identified in our study were analyzed for transcription factor binding site over-representation (iii) using a novel chromatin-guided method, REMINISCE (Figure S1 and Methods). REMINISCE leverages macrophage epigenomic and chromatin measurements, such as histone acetylation [38], [39] and DNase I hypersensitive sites [40], in the analysis of the 5′ regulatory regions of differentially expressed genes, including enhancers as well as promoters. In both the *Reversa* and aortic transplant regression models, we found that the consensus binding site sequence for the T-cell specific, HMG-box factors (TCF) and lymphoid enhancer factors (LEF), together known as the TCF/LEF family of transcription factors, was overrepresented within the 5′ regulatory regions of genes that are upregulated in plaque macrophages during regression.

TCF/LEF transcription factors are activated by nuclear β-catenin (CTNNB1) that accumulates in response to activation of the canonical Wnt signaling pathway [41], [42]. The Wnt pathway controls multiple functions in development, tissue organization, cell proliferation, cell migration [42], and inflammation [43]. In macrophages, canonical Wnt signaling is thought to promote cell motility through a β-catenin-dependent mechanism [44]. In this study we show, for the first time, that activation of canonical Wnt signaling, defined by up-regulation of β-catenin, occurs within macrophage-rich plaque in two mechanistically distinct lipid-lowering models of plaque regression, the *Reversa* and aortic transplant models.

## Results

### Identification of pathways and gene functions that are associated with plaque macrophage responses to lipid lowering *in vivo*

**Figure 1:**
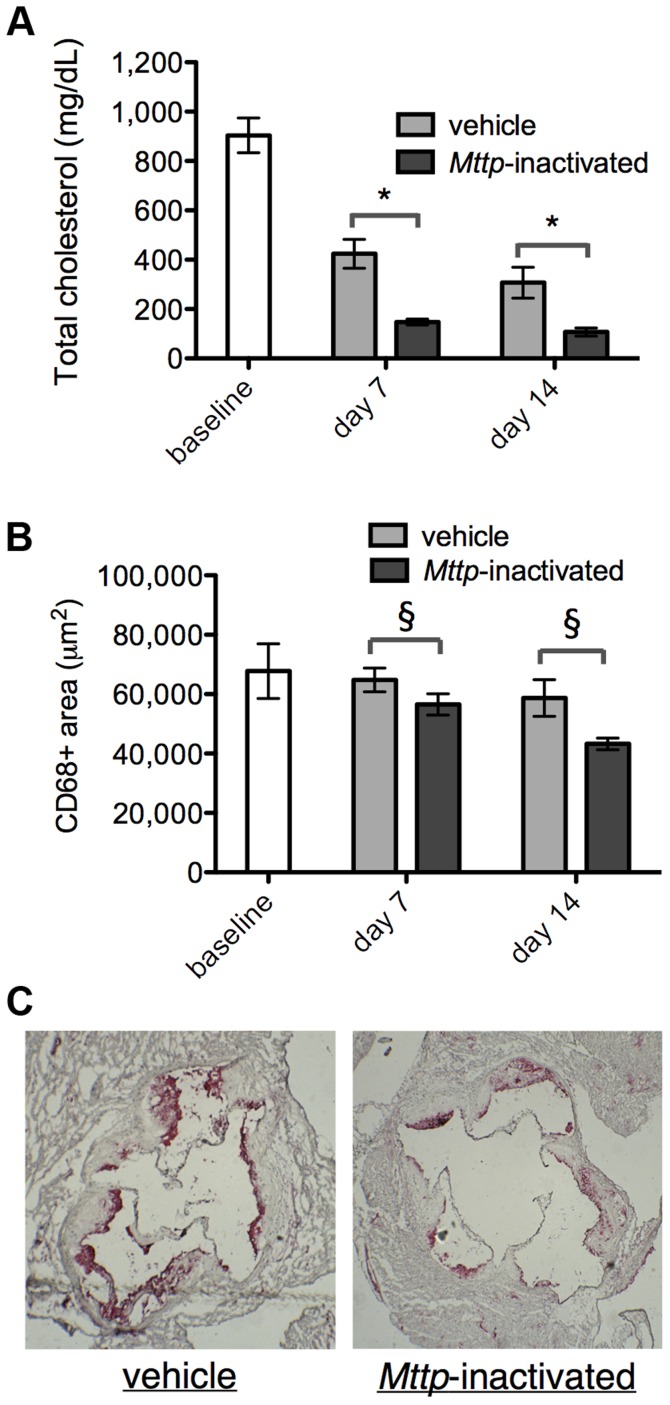
Reversal of hyperlipidemia in *Reversa* mice reduces plaque macrophage content. (A) *Plasma cholesterol levels were reduced upon switching to chow diet and upon inactivation of Mttp*. Bars are mean ± SE. Cholesterol differences between vehicle and *Mttp*-inactivated are: at day seven, 65% reduction; at day 14, 65% reduction. The cholesterol reduction in *Mttp*-inactivated vs. vehicle, at days seven and 14, is significant with *P*<10^−4^ [(*) two-way analysis of variance (ANOVA)]. For all bars, *N* = 5 except for baseline, where *N* = 7. (B) *Mttp inactivation contributes to a reduction in macrophage content of the plaque*. Bars are mean ± SE. CD68+ area differences between vehicle and *Mttp*-inactivated are: at day seven, 13%; at day 14, 26%. The CD68+ area reduction in *Mttp*-inactivated vs. vehicle groups, at both days seven and 14, is significant with *P*<0.05 (§, two-way ANOVA). For all bars, *N* = 7 except day seven, where *N* = 9. The difference in CD68 areas between day 14 (or day seven) saline-treated groups and baseline were not statistically significant. (C) *Mttp inactivation reduces CD68 cellular content within aortic root plaque*. Micrographs are representative aortic root sections from day 14 animals (from vehicle and *Mttp*-inactivated groups, as indicated) that have been counterstained with hematoxylin (blue) and immunostained for CD68 (red).

*Reversa* mice were maintained on Western diet for 16 weeks, which allowed for the development of hypercholesterolemia (903±71 mg/dL total cholesterol [mean ± standard error, SE]) and substantial, macrophage-rich plaques at the aortic root, as determined by immunostaining with the macrophage marker Cluster of Differentiation 68 (CD68). At 16 weeks, a group of animals were sacrificed to measure the baseline plasma total cholesterol levels and CD68+ areas within plaque within aortic root sections. The remaining animals were switched to chow diet and divided into two groups, an experimental group that received four injections of polyinosinic:polycytidylic acid (poly I:C) to induce Mx1:*Cre* in order to inactivate *Mttp^fl/fl^*, and a control group that was injected with vehicle (saline) on the same schedule. On day seven or 14 after the final injection, animals were sacrificed and plasma cholesterol and plaque CD68+ areas were measured. By day 14, cholesterol levels had decreased by 88% in the *Mttp*-inactivated animals vs. baseline (Figure 1A and Table 1, *P*<0.001) and plaque CD68+ area had decreased by 36% in the *Mttp*-inactivated animals vs. baseline (Figure 1B and Table 2, *P*<0.05). Additionally, plaque CD68+ area was reduced in *Mttp*-inactivated vs. vehicle-treated animals at seven and 14 days (*P*<0.05); at day 14, CD68+ area was 26% smaller in the *Mttp*-inactivated animals to the vehicle-treated animals (Figure 1C). These results indicated that there is plaque regression that is attributable to inactivation of *Mttp*. In contrast, comparisons of the saline-treated sample groups to baseline showed no statistically significant differences in CD68+ area that could be attributed to diet switch alone.

**Table 1:** *Mttp* inactivation lowers plasma cholesterol in the treatment vs. control groups.

Group	Number of animals	Plasma total cholesterol level (mg/dL)
baseline	7	903±71
vehicle, day 7	5	424±59
*Mttp*-inactivated, day 7	5	148±12
vehicle, day 14	5	307±62
*Mttp*-inactivated, day 14	5	107±16

*Reversa* mice were fed a Western diet for 16 weeks. One group of animals was sacrificed at 16 weeks as the baseline group. The rest of the mice were switched to chow diet and assigned to two groups, vehicle and *Mttp*-inactivated; animals within these two groups received injections of saline or poly I:C, respectively (see Methods). Animals were sacrificed on day seven or day 14 after the injections, and plasma total cholesterol levels were measured (see Methods). Measurements are summarized as mean ± SE.

**Table 2:** *In vivo* lipid lowering leads to reduced aortic root CD68+ area, independent of diet switch.

Group	Number of animals	Area of CD68+ cells within aortic root sections (µm^2^)
baseline	7	67,800±9,200
vehicle, day 7	9	64,800±4,000
*Mttp*-inactivated, day 7	9	56,500±3,600
vehicle, day 14	7	58,700±6,200
*Mttp*-inactivated, day 14	7	43,300±2,000

*Reversa* mice were fed a Western diet for 16 weeks. One group of animals was sacrificed at 16 weeks as the baseline group. The rest of the mice were switched to chow diet and assigned to two groups, vehicle and *Mttp*-inactivated; animals within these two groups received injections of saline or poly I:C, respectively (see Methods). Animals were sacrificed on day seven or day 14 after the injections, and aortic roots were sectioned, immunostained for CD68, and the CD68+ areas were measured (see Methods). Measurements are summarized as mean ± SE.

In order to focus on the molecular signaling events that initiate the reduction in plaque macrophages, we selected an early time point (day seven) when there was a statistically significant difference in the CD68+ area in the treatment group vs. vehicle (Figure 1, B and C). CD68+ plaque macrophages were isolated from aortic root sections from *Mttp*-inactivated *Reversa*, vehicle-treated *Reversa*, and poly I:C-injected *Ldlr* ^−/−^ animals (Table S1) by laser capture microdissection (LCM) [45], [46], a procedure that we have previously demonstrated specifically enriches for the mRNAs of macrophage-related genes by more than 30-fold over levels seen in aortic homogenates [45]. Global transcriptome profiles were then obtained from the LCM-derived samples using high-density oligonucleotide microarray hybridization. A total of 213 genes were detected as differentially expressed in plaque macrophages from *Mttp*-inactivated vs. vehicle-treated animals (93 had higher expression levels in *Mttp*-inactivated than in control, and 120 had lower levels; Table S2). In order to control for any effect of the poly I:C injections on the macrophage transcriptional profile we compared the transcriptomes of plaque CD68+ cells from saline-treated *Reversa* mice with those from poly I:C-treated *Ldlr* ^−/−^ mice. We found that only one of the 213 lipid-responsive genes, *Trnt1*, was detected as differentially expressed in the *Reversa* saline vs. *Ldlr* ^−/−^ poly I:C comparison, and this gene was excluded from further bioinformatic analysis. To further substantiate that the 213 genes in plaque CD68+ cells are responding to lipid lowering rather than directly to poly I:C, the list of 213 genes was compared with a list of genes that are differentially expressed (vs. vehicle) in bone marrow-derived macrophages stimulated *in vitro* with poly I:C (Table S3) to determine whether the degree of overlap in the two gene lists is greater than would be expected by chance. The analysis revealed that there is not more overlap with *in vitro* poly I:C responses than would be expected by chance (*P*>0.1, Fisher's Exact Test).

**Figure 2:**
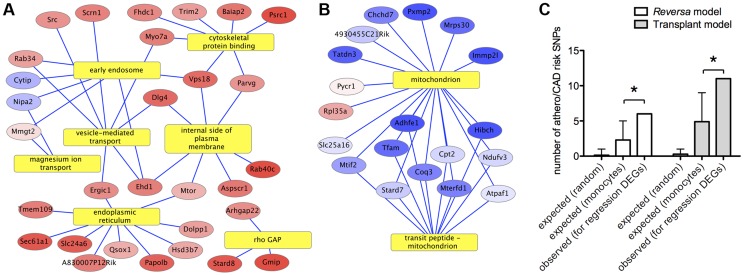
Functional bioinformatic analysis of genes that respond to lipid lowering *in vivo* indicates connections with cell motility and risk of human CAD/atherosclerosis. Yellow rectangles represent gene annotation categories. Ellipses represent genes that are differentially expressed between *Mttp*-inactivated and vehicle-treated sample groups in plaque macrophages in the *Reversa* mouse model of plaque regression (red = up in *Mttp*-inactivated, blue = down). Genes are labeled by official gene symbol. Color saturation indicates the magnitude of the fold-change (see Table S2). Edges connect genes to the statistically enriched annotation categories with which they are associated (see Methods; enrichment *P* values are given in Table S4). (A) *A statistically significant cluster of upregulated genes is associated (by gene annotations) with the cytoskeleton*. (B) *A statistically significant cluster of downregulated genes is associated with the mitochondrion*. (C) *Human monocyte expression QTLs for genes that are differentially expressed in plaque CD68+ cells in regression are enriched for SNPs associated with risk of atherosclerosis or CAD, versus the number of atherosclerosis/CAD SNPs for monocyte eQTLs overall*. The bars marked “expected” show the range of numbers of SNPs associated with risk of atherosclerosis or CAD that would be expected to occur by chance within the eQTLs of the set of 213 lipid lowering-responsive genes, based on the overall percentage of SNPs that are associated with risk of atherosclerosis/CAD (“random”) and based on the percentage of SNPs within all monocyte eQTLs that are associated with atherosclerosis/CAD (“monocytes”). Error bars indicate >90% confidence intervals for the number of atherosclerosis/CAD SNPs that would be expected by chance (see Methods). (*) *P*<0.05, Fisher's Exact Test (contingency table data are in Table S6).

To gain insight into the biological processes that occur during lipid lowering *in vivo*, we performed gene functional annotation enrichment analysis. The most significantly overrepresented processes associated with the upregulated genes are related to cell motility, cytoskeletal binding, and Rho GTPases (Figure 2A and Table S4). For the genes that were downregulated in macrophages from lipid-lowered vs. control animals, the gene annotation term that was the most significantly overrepresented was mitochondrion (Figure 2B and Table S4). In order to determine whether lipid lowering *in vivo* might have increased plaque macrophage apoptosis, multiple gene functional enrichment analysis tools were applied, and in each case, no finding of a statistical enrichment for apoptosis-related gene annotations was detected (see Methods).

### Contextualization of lipid lowering-responsive genes with genes identified in previous studies and atherosclerosis risk alleles in humans

We hypothesized that among the plaque macrophage genes that are responsive to lipid lowering *in vivo*, genes that had been previously reported to be differentially expressed in response to lipid loading *in vitro* would be statistically overrepresented. We therefore combined differentially expressed gene lists from 21 datasets from 15 studies in the atherosclerosis and macrophage foam cell literature (Table S5), and scored genes based on the number of studies in which they were detected as differentially expressed during foam cell formation or disease progression (similar to the approach that was used in a recent meta-analysis to identify obesity-related genes [47]). We found that 19 out of the 213 differentially expressed genes in our study were also detected among the high-scoring genes in the *in vitro* lipid-loaded macrophage meta-analysis gene set, corresponding to a 2.9-fold enrichment (*P*<0.0001) vs. for all mouse genes.

Because *in vivo* lipid lowering is associated with plaque regression in the *Reversa* and aortic transplant models (Figure 1 and [19], [20], [23], [36]), we conjectured that the gene expression changes in plaque CD68+ cells in response to lipid lowering would be associated with atheroprotective effects in humans. Because such effects might be subtle for any individual gene, and because “master regulators” of the transcriptional response are often not transcriptionally regulated themselves, we tested our hypothesis by computationally searching for connections between human atherosclerosis/CAD risk-associated single nucleotide polymorphisms (SNPs) and monocyte eQTLs for orthologs of genes that are differentially expressed in plaque CD68+ cells in response to lipid lowering (as had been previously applied to a transcriptome study of whole aortic arches [30]). We assumed that if there are no atheroprotective effects associated with the observed gene expression changes in CD68+ cells, then the fraction of atherosclerosis/CAD risk SNPs within monocyte eQTLs for the 213 lipid lowering-responsive genes should be the same as the fraction of atherosclerosis/CAD SNPs within all monocyte eQTLs. Using a list of human eQTLs from two population genetic studies of peripheral blood monocyte gene expression [48], [49], we identified SNPs associated with mRNA levels (eSNPs) of genes whose mouse orthologs are differentially expressed in CD68+ cells during regression in the two mouse models. We expanded the regression-gene-set-associated eSNPs to include all validated SNPs in linkage disequilibrium (using haplotype information from the 1,000 Genomes Project [50]), obtaining a set of regression-associated monocyte eQTLs. We then assessed the frequency of atherosclerosis/CAD risk SNPs (starting with a list of 761 risk SNPs obtained from the NCBI PheGenI database [51]) within these regression-associated monocyte eQTLs and compared these frequencies to the frequency of atherosclerosis/CAD risk SNPs within monocyte eQTLs in general. We found that the regression-associated eQTLs are substantially more enriched for atherosclerosis/CAD risk SNPs than monocyte eQTLs in general (Figure 2C and Table S6), in both regression models (*P*<0.05 for each model); overall, a total of 17 atherosclerosis/CAD risk-associated SNPs were found within regression-associated eQTLs (Table S7). Further investigation revealed that one of the *Reversa* regression-associated eQTLs encompasses rs599839, a SNP in the 1p13.3 locus that has been found to be associated with LDL cholesterol (LDL-C) levels and with risk of CAD and MI [52]–[55], as well as with hepatic mRNA expression of the intracellular lipoprotein sorting receptor Sortilin 1 (*SORT1*) [56], [57]. In human monocytes [48] and in liver cells [56], variant rs599839 is also an eSNP for mRNA expression of the gene Proline/serine-rich coiled coil 1 (*PSRC1*), which is one of most strongly upregulated genes in CD68+ cells in *Mttp*-inactivated vs. vehicle-treated *Reversa* mice (Table S2).

### Identification and targeted validation of transcriptional regulators that are associated with macrophage responses to lipid lowering *in vivo*

**Figure 3:**
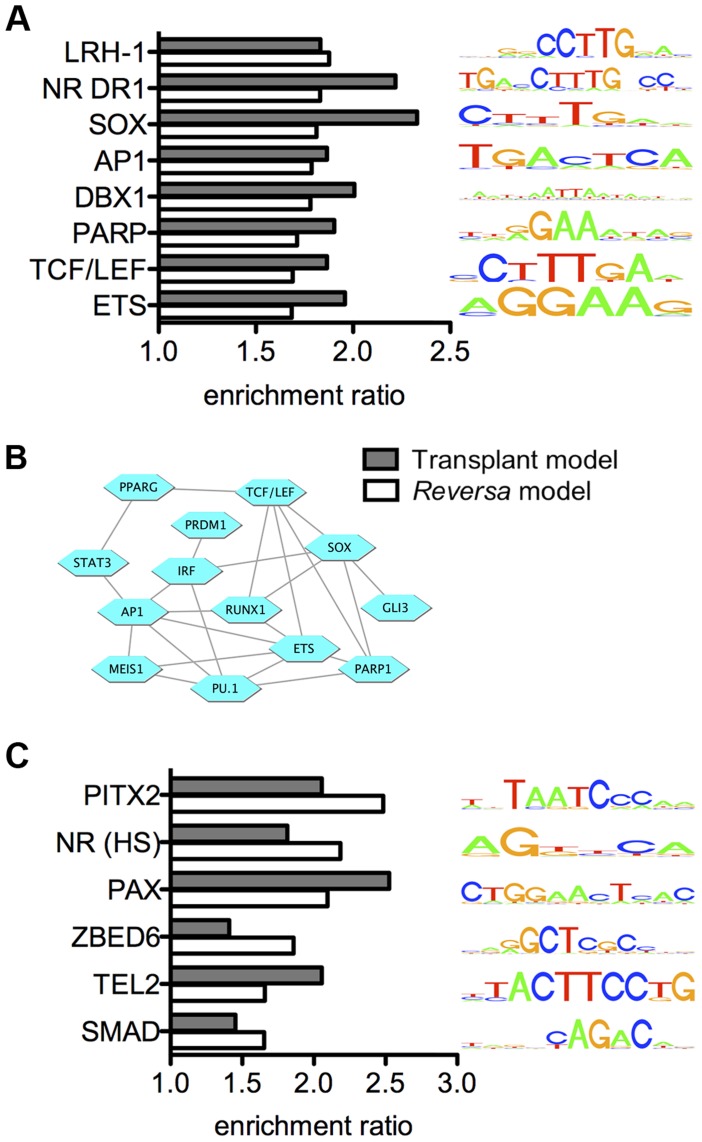
Integrated analysis reveals candidate regulators of the macrophage transcriptional response to lipid lowering *in vivo*. (A) *Transcription factor binding site motifs whose matches are statistically overrepresented within 5′ regulatory regions of genes that are upregulated in plaque macrophages in response to lipid lowering in vivo*. Bars indicate the ratio of the number matches of the indicated motifs per kbp, within an upregulated gene set, to the number of motif matches per kbp for randomly selected sets of genes expressed in CD68+ cells. Bar color indicates the regression model: white, *Reversa* model; gray, aortic transplant model. Only motifs for which the ratio exceeds 1.5 (with a statistical significance of *P*<0.01) in *both* models are shown (complete list in Table S9). Motifs are labeled by the transcription factor family name with which they are associated (motif IDs are provided in Table S9). Each motif is represented by a four-color motif logo. (B) *The transcription factors that are associated with upregulated genes are highly interconnected at the level of protein-protein interaction network*. Blue hexagons represent transcription factors or transcription factor families, and edges denote protein-protein interactions between them. (C) *Transcription factor binding site motifs whose matches are statistically overrepresented within 5′ regions of genes that are downregulated in plaque macrophages in response to lipid lowering in vivo*. “NR (HS)”, nuclear receptor half-site.

We applied our novel promoter scanning analysis method, REMINISCE (Figure S1), to identify candidate transcription factors that are associated with the transcriptional responses of plaque macrophages to lipid lowering *in vivo*. REMINISCE combines 17 different types of macrophage chromatin and epigenomic measurements (see Table S8) that were obtained from NCBI GEO and from the Mouse ENCODE project [58] in a machine-learning algorithm (using genome regions that coincide with transcription factor binding sites that are obtained from ChIP-seq as a training dataset of regulatory regions) to map macrophage *cis*-regulatory regions, and then scans genomic sequences within these regions to identify statistically overrepresented transcription factor binding site sequence patterns (see Methods). In a direct comparison of REMINISCE with non-epigenome-guided motif-scanning of all noncoding sequence within ±1 kbp or ±5 kbp of the transcription start site for gene sets derived from macrophage transcriptional responses to the atherogenic lipoprotein oxidized LDL, REMINISCE detected substantially more binding site motifs above the significance threshold (Figure S2). Applying REMINISCE to the 213 lipid lowering-responsive genes from the *Reversa* model, we identified 15 transcription factor binding site sequence motifs that are statistically overrepresented within the 5′ regulatory regions of upregulated genes (Figure S3A), and 14 motifs that were associated with the downregulated genes (Figure S3B). Because any experimental model is subject to model-specific artifacts, we also analyzed our previously published data on the transcriptional response in CD68+ cells isolated from regressing plaques in the aortic transplant model using the REMINISCE method. We found that of the 15 motifs that were overrepresented among upregulated genes in the *Reversa* model, eight were also overrepresented among upregulated genes in the aortic transplant model (Figure 3A), including the motifs for PPARγ (nuclear receptor direct repeat 1, or NR DR1), the TCF/LEF family of transcription factors, and activating protein 1 (AP-1). The transcription factors represented by the 15 motifs that were associated with upregulated genes in the *Reversa* model are highly interconnected in the human protein-protein interactome [59] (clustering coefficient of 0.31, vs. 0.14 for the whole interactome [60]) (Figure 3B). Similarly to the case with transcription factors for upregulated genes, of the 14 motifs that were overrepresented among downregulated genes in the *Reversa* model, six were also detected in the aortic transplant model (Figure 3C and Table S9).

**Figure 4:**
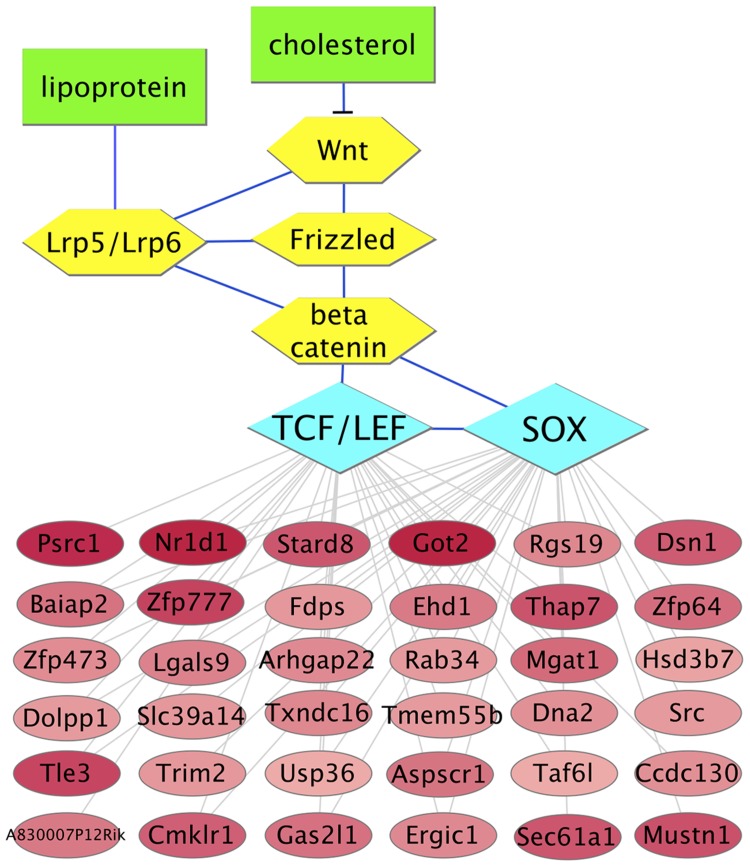
Predicted molecular interaction network for upregulated genes that possess CTTTGA elements connects cholesterol to motility. Red ellipses represent genes whose expression levels are elevated in plaque macrophages from *Mttp*-inactivated vs. vehicle-treated animals (darker color indicates a greater differential expression ratio; see Table S2). Yellow hexagons and blue edges represent the upstream signaling pathway based on the literature (the blunt-ended edge indicates inhibition of signaling), with green rectangles representing lipids (*cholesterol*: membrane cholesterol; *lipoprotein*: apoB-containing lipoprotein). Blue diamonds represent transcription factor binding site motifs. Gray edges connect the transcription factors to the downstream genes that possess corresponding binding site sequence matches.

Under the hypothesis that the transcription factors that control macrophage responses to lipid lowering *in vivo* would have binding site sequences within the 5′ regulatory regions of a high proportion of differentially expressed genes, we ranked the motifs from Figure 3 by the fraction of the differentially expressed genes that possess a match for each motif (Table S10). Notably, for the upregulated genes, the motif whose matches were present within the largest percentage of genes, 33%, was the CTTTGA motif that is recognized by TCF/LEF and SOX transcription factor families. The finding of enrichment of TCF/LEF binding site sequences within 5′ regulatory regions of genes that are upregulated in the *Reversa* mouse was confirmed by additional computational analysis using high-precision motifs based on three-dimensional structural modeling of protein-DNA interactions [61] (Table S9). Because the reduced macrophage content of the plaque in the transplant regression model has been associated with macrophage emigration from the plaque, we also ranked transcription factor binding site motifs based on the proportion of cytoskeletal-binding and RhoGAP domain-containing genes (Table S4) whose 5′ regulatory regions contain matches for each motif. We found that the motif with the highest percentage, 50%, was the CTTTGA motif (corresponding to TCF/LEF and SOX) (Table S10). Next, we used the global mammalian protein interaction network to examine which signaling pathways could connect between lipoproteins or cholesterol and these transcription factors. We found that the Wnt signaling pathway [62], [63], through β-catenin and through TCF/LEF and SOX family members [64], satisfies these criteria (Figure 4).

Gene regulation likely occurs at multiple levels in plaque macrophages. Therefore, we scanned the 3′ untranslated regions (UTRs) of the two sets of differentially expressed genes in plaque CD68+ cells in the *Reversa* model for microRNA target sequences. We identified eighteen microRNAs whose target sequences were overrepresented within the 3′ UTRs of the upregulated and downregulated genes (six for upregulated genes, and twelve for downregulated) (Figure S4, Table S11). Under the hypothesis that some of these microRNAs might work in concert in response to lipid lowering *in vivo*, we analyzed molecular pathways for enrichment of the number of pathway-associated genes that possess target sequences for any of the microRNAs in Figure S4A, using a microRNA pathway analysis tool [65]. For the upregulated genes, the two pathways with the strongest enrichment were axon guidance (*P*<10^−4^) and focal adhesion (*P*<0.001); also enriched was the Wnt signaling pathway (*P*<0.05) (Table S12).

**Figure 5:**
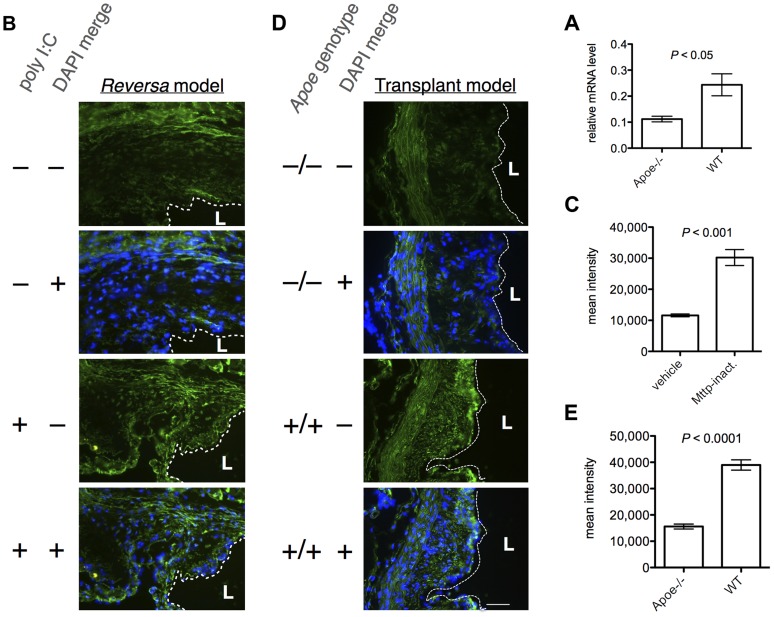
β-catenin expression is increased in regressing vs. progressing plaques, in both models of plaque regression. (A) The relative *Ctnnb1* (β-catenin mRNA) *levels, as measured by qPCR, are higher in CD68+ cells in regressing plaques than in progressing plaques, in the aortic transplant model (day five post-transplant)*. Bars, mean ± SE (*N* = 6 animals per sample group). (B, C) *Plaques in Mttp-inactivated Reversa mice have higher levels of β-catenin than plaques in vehicle-treated animals*. (B) Representative fluorescence micrographs from an immunohistochemical analysis of β-catenin expression at the aortic root in the *Reversa* mouse at day seven post-injection to inactivate *Mttp* (*N* = 3 animals per group, six sections analyzed per animal). Green, β-catenin immunofluorescence; blue, diamidino-2-phenylindole (DAPI) fluorescence; dashed white line divides lumen (“L”) from intima; scale bar, 50 µm. The green signal in the upper part of the top panel is autofluorescence from the elastic lamina. (C) Plaque β-catenin expression for each treatment group was quantified by morphometric image analysis. Bars, mean ± SE (*N* = 4 per treatment group); “Mttp-inact.”, *Mttp*-inactivated. (D, E) *Plaques in WT recipient animals have higher levels of β-catenin than do plaques in Apoe^−/−^ recipients, in the transplant regression model*. (D) Representative fluorescence micrographs from an immunohistochemical analysis of β-catenin expression in the aortic transplant model (*N* = 3 animals in each genotype group, six sections per graft), at day five post-transplant. Color channels and annotations are as for (B). (E) Plaque β-catenin expression for each genotype group, by morphometric analysis. Bars, mean ± SE (*N* = 4 per genotype group). *P* values in (A,C,E) are from unpaired, two-tailed, Student's *t*-tests.

β-catenin-dependent activation of TCF/LEF is the final step in the canonical Wnt signaling pathway. To investigate the possibility of canonical Wnt pathway activation during plaque regression, we measured the relative mRNA level of β-catenin (*Ctnnb1*) in CD68+ cells isolated from aortic grafts from WT and *Apoe* ^−/−^ recipient mice in the aortic transplant regression model. By quantitative PCR (qPCR), we found that the relative level of *Ctnnb1* mRNA is approximately 2.2-fold higher in CD68+ cells in regressing plaques than in progressing plaques (Figure 5A) (*P*<0.05). Additionally, we measured mRNA levels of two known Wnt target genes, *Lrp6* (for which loss-of-function has been associated with premature CAD [66]) and *Gja1* [67], both of which were indicated by microarray profiling to be upregulated in regressing vs. progressing plaque CD68+ cells in the aortic transplant model [36]. By qPCR, both *Lrp6* and *Gja1* were found to be upregulated in CD68+ cells in regressing vs. progressing plaques (Figure S5). Given a previous report that β-catenin deficiency impairs macrophage migration [44] and in light of evidence of plaque macrophage emigration during regression in both the *Reversa* [20] and aortic transplant [22] models, we investigated whether canonical Wnt pathway activation stimulates macrophage migration *in vitro*. In a scratch-wound cell migration assay [68], [69], primary murine macrophages treated with the canonical Wnt pathway agonist Wnt3a scored more than two-fold higher than vehicle-treated macrophages (Figure S6).

To confirm the prediction that Wnt signaling is activated in plaque macrophages during regression *in vivo*, we analyzed the expression of β-catenin in plaque within aortic root sections from *Reversa* mice at day seven following *Mttp*-inactivation or vehicle treatment, by fluorescence immunohistochemistry. Consistent with the systems biological analyses, the level of β-catenin was significantly greater (approximately 2.6-fold, *P*<0.001) in the plaques isolated from the *Mttp*-inactivated vs. vehicle-treated animals (Figure 5B, 5C). Because the TCF/LEF binding site was detected as being overrepresented among genes that are upregulated during plaque regression in the transplant regression model as well, we also analyzed β-catenin expression in plaques in the aortic arch graft of WT and *Apoe* ^−/−^ recipient animals. As with the *Reversa* model, we found that β-catenin is expressed at an approximately 2.5-fold higher level (*P*<0.0001) in regressing vs. progressing plaques (Figure 5D, Figure 5E, Figure S7), with increased abundance in the nuclear area (Figure S8).

## Discussion

It is clear that atherosclerosis regression involves the interactions of many cellular pathways and multiple cell types and plaque components. To address this complexity, a systems biology approach employing large-scale molecular profiling is needed to identify core, model-independent mechanisms [8], [10], [70], [71]. To our knowledge, the present work, along with complementary measurements obtained from the aortic transplant regression model [36], represent the first reports of transcriptome measurements specifically of CD68+ macrophages within regressing plaques. In a previous transcriptome study [29] of changes in vessel wall tissue after lipid lowering *in vivo* in the *Reversa* mouse, a modest number of genes were detected as differentially expressed in the aortic wall (37 genes in [29], and 42 genes for arteries containing early lesions in [30]). Notably, none of these was detected as differentially expressed in our study of responses of plaque macrophages to lipid lowering, pointing to the need to separately measure the gene expression changes of constituent cell types of the plaque [71], as we have done for macrophages in the present work. While the 213 genes we identified as differentially expressed in plaque CD68+ cells in the *Reversa* mouse in response to lipid lowering are likely only a subset of the transcriptional changes in plaque macrophages over various time points, the enrichment of atherosclerosis/CAD risk alleles within human monocyte expression QTLs for orthologs of these genes (Figure 2C) provides intriguing support for their collective function in macrophages in the context of atherosclerosis.

Given the central role of macrophages in vascular inflammation [72] and in plaque destabilization in humans [73]–[75], our finding that *Mttp*-inactivation in the *Reversa* mouse reduces the macrophage content of the plaque (Figure 1 and [20]) points to the potential for beneficial remodeling. However, it also raises the question of how much of this reduction is driven by three processes (all of which have been reported in different models of regression): emigration from the plaque [22], deceased infiltration of monocytes [17], and increased macrophage cell death [25]. To the extent that accumulation of apoptotic macrophages within shoulder regions of advanced plaque is considered a marker of vulnerable plaques [76], the balance of these mechanisms is potentially clinically significant.

In the aortic transplant regression model, plaque macrophages in WT recipients rapidly emigrate from the graft to regional lymph nodes [22], while remaining macrophages do not exhibit abnormal levels of apoptosis [22]. Measurements of macrophage transcriptional changes during plaque regression versus progression in the aortic transplant model are consistent with an induced emigration hypothesis; macrophages in regressing (vs. progressing) plaques had lower expression levels of genes associated with adhesion and higher expression levels of genes associated with cell motility [36]. In regards to the emigration hypothesis in the *Reversa* model (which is supported by macrophage trafficking studies *in vivo* [20]), the plaque macrophage transcriptome measurements in this work provide several key insights. First, among genes that are upregulated in macrophages from *Mttp*-inactivated versus control animals, genes encoding cytoskeletal-binding proteins and Rho GTPases are overrepresented, consistent with increased motility. Second, among genes that are differentially expressed, no overrepresentation of apoptosis-related genes was found at the time point that we examined. A third insight, involving the Wnt signaling pathway, comes from the transcription factor analysis as described below.

In this study, we used an integrative computational approach, REMINISCE, to identify transcription factors that are likely to mediate macrophage responses during plaque regression. A novel aspect of the approach is that it incorporates macrophage epigenome measurements to guide the search for transcription factor binding sites (resulting in improved sensitivity; Figure S2) in the context of an enrichment analysis. In the analysis, we made use of transcriptome measurements from both the aortic transplant and *Reversa* regression models as well as macrophage-specific chromatin measurements to detect overrepresentation of known transcription factor binding site sequences upstream of genes that are differentially expressed during plaque regression. To focus on transcription factors that may have a model-independent role in macrophage responses to lipid lowering, we selected factors whose binding site sequences were overrepresented in *both* regression models. The significant overlap between the sets of transcription factors that were identified from the two models was unexpected due to the distinct differences in genetic backgrounds, plaque locations, and regression time-scales of the models. Of these factors, the association of PPARγ with genes that are upregulated during plaque regression is consistent with one of the principal findings from a previous transcriptome profiling study of whole aortic arches in the *Reversa* mouse with early lesions [30]. While our previous work investigating the effect of treatment of *Reversa* mice with a PPARγ agonist did not observe a significant effect of the agonist on the abundance of macrophages in the plaque [20], PPARγ is certainly worthy of future studies to clarify its precise function in plaque regression (especially given conflicting findings in different models and disease stages [20], [77]–[79]).

Among the other transcription factors whose binding site motifs were found to be associated with genes that are upregulated in CD68+ cells in regressing plaques in both regression models, the TCF/LEF and SOX transcription factor families (whose binding site sequences were associated with genes that are upregulated in response to lipid lowering) were particularly notable because of their connection to Wnt signaling, and thus, cell motility. Additionally, pathway enrichment analysis based on genes with target sequences for the microRNAs that were associated with upregulation in plaque CD68+ cells identified axon guidance and Wnt signaling as enriched pathways. Based on the transcription factor binding site analysis, we hypothesized that canonical Wnt signaling may be increased in plaque macrophages during regression. This hypothesis was confirmed by multiple observations including *Ctnnb1* mRNA measurements in CD68+ cells, mRNA measurements of Wnt pathway target genes, and immunohistochemical analysis demonstrating increased β-catenin in regressing plaques in the *Reversa* and aortic transplant mouse models. Although the molecular mechanisms that cause increased Wnt signaling in plaque in response to lipid lowering are not known, and, of course, in spite of our associative findings, it is possible that plaque regression is mediated by Wnt-independent pathways, nonetheless, the observation of increased Wnt signaling in response to lipid lowering is consistent with previous reports that, in cultured cells, depletion of cholesterol increases canonical Wnt signaling [63].

In both the *Reversa* and aortic transplant models of regression, lipid lowering *in vivo* leads to decreased macrophage content in plaque (Figure 1B, [20], [23], [28]–[30], [36]), and trafficking studies in both of these models suggest that lipid lowering triggers macrophage emigration from the plaque [20], [22]. Our own functional studies of macrophage responses to Wnt3a stimulation (Figure S6) as well as previous studies of the role of β-catenin in macrophage migration [44], [80], are consistent with the model that increased canonical Wnt signaling – resulting in increased β-catenin levels – stimulates macrophage motility. A transitioning of macrophages from a sessile to a motile state would be expected to facilitate macrophage egress from the plaque [81].

This work is the first to our knowledge to propose that canonical Wnt signaling is involved in atherosclerosis regression in response to lipid lowering *in vivo*, and adds to several lines of evidence that support that alterations in Wnt signaling play a role in the *progression* of atherosclerosis. *LRP6* genetic variants that impair Wnt/β-catenin signaling have been reported that are associated with increased risk of carotid atherosclerosis [82] and early CAD [66] in humans, suggesting that decreased canonical Wnt/β-catenin signaling may be pro-atherogenic; conversely, by this reasoning, increased Wnt signaling would be expected to have protective, pro-regressive effects. Consistent with this model, the canonical Wnt signaling molecule Wnt3a has been demonstrated to mediate anti-inflammatory effects via β-catenin-dependent suppression of the expression of proinflammatory cytokines [83]. Canonical Wnt signaling is also thought to play an important role in maintaining vascular cellular homeostasis, for example, in orchestrating lineage commitment within pericytes in the vessel wall [84]. Furthermore, Wnt signaling has been shown to potently inhibit lipid accumulation in cultured cells and to regulate plasma cholesterol levels *in vivo* [85]–[87], as well as (noted before) to promote macrophage migration ([44] and Figure S6).

While the present work provides evidence implicating β-catenin-interacting transcription factors in atherosclerosis regression, it is highly probable that other transcription factors are involved, given the complexity of macrophage transcriptional regulation in response to changing lipoprotein levels [70]. Of particular interest for future studies are the transcription factors that have overrepresented binding site sequences within the upregulated gene group, and that are connected to the TCF/LEF family members in the protein interaction network (Figure 3B). It should also be noted that a potential limitation of the analytical approach used in this work is the partial reliance on proximity to key factors in the global protein interaction network, in prioritizing transcriptional regulators for targeted validation; the use of such a proximity criterion could introduce bias against transcription factors that are less well-studied.

While this work used curated chromatin measurements from primary macrophages to guide the bioinformatic search for transcription factor binding sites, the numbers of cultured cells that are required for a ChIP-seq assay is rapidly being reduced; continued assay improvements may make possible genome-wide location analysis of chromatin factors within specific cellular constituents of plaque, which would likely enable more precise mapping of regulatory elements that mediate transcriptional changes during plaque regression. Equally important, comprehensive datasets of genetic variants and their associations with cardiovascular phenotypes (which may achieve significance only in a meta-analysis), such as those provided by the CARDIoGRAMplusC4D and dbGAP databases, will undoubtedly be beneficial for contextualizing the functions of these regulatory elements in atherosclerosis. Another enhancement in future studies would be to use lineage-tracing techniques *in vivo* to definitively characterize the extent of cellular phenotypic conversion in the *Reversa* model at the aortic root. Our previous work, however, has demonstrated that CD68-guided LCM of aortic root plaque yields a highly macrophage-enriched cell population [45]. Furthermore, no transcriptional changes were observed during plaque regression that would suggest a change in the proportion of macrophages in the cells captured by CD68-guided LCM in regressing vs. progressing plaques.

Beyond the enrichment of TCF/LEF transcription factor binding sites, the finding of enrichment of binding sites for the oxidative stress-responsive transcription factor nuclear respiratory factor 2 (NRF-2) within the regulatory regions of the downregulated gene group (Figure S3) is interesting in light of the observed down-regulation of mitochondrial transcription factor A (*Tfam*, a regulator of mitochondrial biogenesis and a known NRF target gene [88]). Downregulation of NRF-2 activity might reflect decreased oxidative stress in plaque macrophages due to the substantially reduced plasma lipoprotein levels [89].

Another intriguing finding from our work is the higher level of expression of *Psrc1* in plaque macrophages in the *Reversa* regression model. *Psrc1* mRNA has been previously been reported to be elevated in macrophage-rich regions of human atheroma [90]. The function of PSRC1 in macrophages is not known, but in other cell types it stabilizes microtubules [91], [92] and functions in cell migration [92]. Given our findings of elevated β-catenin in regressing plaques, it is interesting that a study of PSRC1 function found that PSRC1 stimulates the β-catenin pathway [93]. While strong evidence indicates that the effect of SNP rs599839 on LDL-C is mediated by hepatic expression of Sortilin 1 (*SORT1*) [56], the link between rs599839 and monocyte expression of *PSRC1* seems worthy of further study in the context of plaque regression.

The multi-model analyses of plaque regression we have carried out is likely to ultimately shed light on the process of plaque stabilization in humans, given that atherosclerosis in genetically modified mice recapitulates many features of clinical disease. Large clinical trials have shown that in humans treated with lipid-lowering drugs, plaque area, as assessed by intravascular ultrasound (IVUS), changes very little [94], [95], while atherothrombotic event rates are dramatically decreased [96], [97], presumably due to stabilization resulting from changes in plaque macrophages not detected by IVUS. Indeed, even in mouse models, we have found that plaque size measurements do not always reflect changes in plaque macrophage content because of increased matrix proteins (e.g., [98]). Demonstrating similar phenomena in human plaques awaits improvements in non-invasive imaging. Overall, in human plaques, a drastic reduction in the abundance of macrophages within the plaque would be presumed to be associated with protective remodeling of the atheroma, and given the similarities in the disease process between mice and people, some of the factors and pathways we have identified are likely to be clinically relevant (e.g., Wnt signaling [66]).

In conclusion, this work demonstrates the value of a genomic, systems biology approach for elucidating the cellular pathways and transcriptional regulatory mechanisms that are dysregulated or altered by perturbations *in vivo*. Although the present study is focused on macrophage responses to lipid lowering *in vivo*, our approach of integrating LCM-derived transcriptome measurements with epigenomic and chromatin structural information from primary cell models could be applied to other constituents of the plaque (e.g., smooth muscle cells and endothelial cells) and then ultimately combined in a comprehensive regulatory network. More broadly, with today's panoply of publicly available epigenomic and chromatin structural measurements from a wide array of mouse and human cell types and tissues (e.g., through the ENCODE project), we anticipate that integrative approaches such as the one used in this work could be useful in the regulatory analysis of transcriptome measurements from unique, highly specific cell populations in other *in vivo* contexts.

## Materials and Methods

### Animal work

This study was carried out in accordance with the recommendations in the Guide for the Care and Use of Laboratory Animals (National Institutes of Health), and in accordance with animal use protocols that were approved by the NYU Langone Medical Center Institutional Animal Care and Use Committee (PHS Animal Welfare Assurance Number A3435-01) or the Institute for Systems Biology Institutional Animal Care and Use Committee (PHS Animal Welfare Assurance Number A4355-1). All tissue harvest (except mouse peritoneal macrophage harvest) and all survival surgeries were carried out under ketamine/xylazine-induced anesthesia, and for survival surgeries, animals were carefully monitored post-surgery for signs of pain or distress. For peritoneal macrophage harvest, mice were euthanized by CO_2_ asphyxiation following standard operating procedures established by the Institute for Systems Biology IACUC.

The development of the *Reversa* mouse (JAX Stock Number 004192, *Ldlr* ^−/−^ *Apob* ^100/100^ *Mttp* ^fl/fl^ Mx1:*Cre* ^+/+^ animals on mixed background, in which injection of poly I:C causes silencing of hepatic *Mttp* expression) has been previously described [19]. In this work, for experimental studies of the *Reversa* model of plaque regression, four-week-old *Reversa* mice were weaned onto a Western diet (Catalog no. 100244, 40% kcal from milk fat; Dyets Inc., Bethlehem, PA) for 16 weeks (baseline time point) to establish aortic plaque and then switched to a chow diet and injected intraperitoneally every other day, for a total of four injections, with either 500 µg poly I:C (Sigma) or vehicle (saline). Blood samples were obtained from the retro-orbital plexus at baseline, seven days after the last poly I:C injection, and 14 days post-injection. Animals were sacrificed for arterial tissue collection at either baseline, seven days after the last poly I:C injection, or 14 days post-injection, as indicated in the Results section.

The aortic transplant model of plaque regression has been previously described [21], [23], [31]. In this work, for studies of plaque regression in the aortic transplant model, C57BL/6–*Apoe* ^−/−^ mice [35] were maintained on Western diet for 16 weeks and then the aortic arches were harvested and grafted into the abdominal aortas of *Apoe* ^−/−^ and WT mice. The grafts were harvested at three or five days post-surgery, as indicated in the Results section.

For all experimental data reported in this work, animal cohort sizes are as reported in the corresponding figure captions, table captions, or main article text, except for the microarray transcriptome profiling of plaque macrophages in the *Reversa* model, for which the cohort sizes are described in Table S1.

### Cell culture

All cell culture was performed at 37°C and in the presence of 5% CO_2_.

Bone marrow-derived macrophages (BMDM) were prepared as follows: L929-conditioned medium was prepared by growing L929 cells to confluency in Dulbecco's Modified Eagle Medium (DMEM)+10% (v/v) fetal bovine serum (FBS)+100 I.U./mL penicillin and 100 µg/mL streptomycin (hereafter, 1% penicillin/streptomycin). The media was then replaced with DMEM containing 2% FBS+1% penicillin/streptomycin. Conditioned media was collected every three days, filtered through a 0.22 µm filter and fresh media containing 2% FBS was added. Bone marrow cells were isolated from the femur and tibia of 8–12 week old mice. After red blood cell lysis (RLB, Sigma), cells were plated in Petri dishes in L929-conditioned medium and incubated for seven days. Cells were then serum-starved in 0.2% FBS overnight and treated with 400 ng/mL Wnt3a (R&D Systems, Minneapolis, MN) for 24 h. RNA was isolated from cells using TRIzol (Life Technologies). cDNA synthesis was performed using Verso cDNA kit (Thermo Scientific) and qPCR analysis was performed as described below (see section qPCR).

Peritoneal macrophages were prepared as follows: In each of three separate replicate experiments, resident peritoneal macrophages were isolated from 2–4 female C57BL/6J mice (ages 8–12 weeks) using intraperitoneal lavage as described previously [70]. Peritoneal exudate cells were pooled, resuspended in peritoneal macrophage medium (Roswell Park Memorial Institute 1640 medium plus L-glutamine plus 10% FBS plus 1% penicillin/streptomycin) and then plated into two tissue culture wells at approximately 10^6^ cells/well. After two hours, wells were triple-washed with warm PBS to select for adherent cells. Cells were incubated for 24 h in the presence of 25 µg/mL (by protein content) CuSO_4_-oxidized human LDL (oxLDL, Intracel, Frederick, MD) or vehicle.

### Scratch-wound macrophage migration assay

BMDM (prepared from *N* = 3 mice) were differentiated in six well plates and grown to confluency. Cells were serum-starved in 0.2% FBS overnight and a single scratch wound was made with a plastic p200 tip. Cells were washed with starvation media to remove non-adherent cells and incubated with or without Wnt3a (400 ng/mL) for 24 h. The cells were then fixed in 3.7% formaldehyde, and wound area visualized. The percentages of area covered in 24 h were quantified using NIH ImageJ software. All conditions were done in quadruplicates.

### Blood and tissue analysis

Plasma cholesterol levels were determined by a colorimetric enzymatic assay (Wako Diagnostics, Richmond, VA). Mice were sacrificed at the time points indicated below (see Results) and hearts (for aortic root analysis) or aortic arch grafts were harvested and frozen in Optimum Cutting Temperature (OCT) compound and serial-sectioned at a thickness of 6 µm onto positively charged glass slides (Superfrost Plus, Fisher Scientific, Pittsburgh, PA).

For CD68 immunostaining and laser capture microdissection, slides were hematoxylin-stained, cleared with xylenes, air-dried, and foam cells were identified by light microscopy. Every seventh section was immunostained with a CD68-specific antibody (AbD Serotec, MCA 1957) as previously described [23] and used as a guide slide for laser-capture microdissection for the next six serial sections. Sections were imaged at 40× on a Leica DM4000B microscope. In addition, slides that were used for morphometric analysis were counterstained with eosin and the CD68-immunostained areas were quantified by computer-aided morphometric analysis of digitized images (Image-Pro Plus software v5, Media Cybernetics, Silver Spring, MD). For isolation of CD68+ cells from the plaques, laser-capture microdissection (LCM) was performed with the PixCell II instrument (Arcturus Bioscience) as previously described [45], [46]. All LCM procedures were performed under RNase-free conditions. Student's *t*-test was used for comparisons of CD68+ pixel areas and cholesterol levels for each sample group vs. baseline. A two-way ANOVA was used to test for differences in CD68+ pixel areas and cholesterol levels, between *Mttp*-inactivated and vehicle-treated sample groups at the two time points (seven and 14 days post-injection). In the linear model for the ANOVA, the post-treatment time point (day seven or day 14) was taken as the first factor, and the treatment (poly I:C or saline) was taken as the second factor.

β-catenin immunohistochemistry (IHC) was carried out using frozen tissue sections from aortic grafts from eight animals (*N* = 4 animals per genotype group). Sections were fixed in acetone for 10 min, blocked with DAKO blocking buffer (DAKO, Cat. # X0909) and incubated with β-catenin rabbit polyclonal antibody (Santa Cruz, Cat, # sc7199, 1∶200) overnight at 4°C. As a negative control, sections from *Apoe* ^−/−^ recipient animals were assayed without the primary antibody. Sections were then incubated with FITC-conjugated secondary antibody for one hour, washed and mounted onto slides using VECTASHIELD mounting medium with diamidino-2-phenylindole (DAPI) (Vector Labs, Burlingame, CA). Images at 63× magnification were obtained using a Leica SP5 confocal microscope. Quantification of the mean fluorescent pixel intensity was performed on immunofluorescent images using Image-Pro Plus software v5. Six sections per animal were analyzed. An unpaired, two-tailed Student's *t*-test was used to test for differences in the numbers of pixels that were positive for β-catenin fluorescence, between the sample groups.

### Microarrays

For LCM-derived samples, RNA was isolated from LCM caps using the PicoPure RNA Isolation Kit (Life Technologies) following the manufacturer's instructions. Total RNA was quantified using the NanoDrop spectrophotometer and assayed for quality using the Agilent Bioanalyzer 2100. Single-stranded cDNA was produced in microgram quantities from the RNA template using the Ovation Pico WTA System v2 (NuGEN), with the QIAquick PCR Purification Kit (QIAGEN) used for cDNA purification. The cDNA was analyzed on the Agilent 2100 Bioanalyzer and then fragmented and biotin-labeled using the Encore Biotin Module (NuGEN).

For peritoneal macrophage-derived samples, total RNA was isolated using TRIzol and RNA quality was analyzed with an Agilent 2100 Bioanalyzer. The three independent experiments thus yielded six RNA samples. Sample mRNA was amplified and labeled with the Affymetrix One-Cycle Eukaryotic Target Labeling Assay protocol and reagents. Labeled cDNA was hybridized to the Affymetrix GeneChip Mouse Exon 1.0 ST array using standard protocols and reagents from Affymetrix.

Fragmented, biotin-labeled cDNA was hybridized to the Affymetrix Mouse Exon Array 1.0 ST GeneChip and the GeneChip was washed and stained using the protocol and reagents in the Affymetrix GeneChip Hybridization, Wash, and Stain Kit. The stained GeneChips were imaged using the Affymetrix GeneChip Scanner 3000 and probe intensities were quantify from the scanned images using the Gene Chip Operating Software from Affymetrix. The transcriptome profiling data for this study are available online in the NCBI Gene Expression Omnibus (GEO) database, under accession numbers GSE52482 and GSE58913.

### qPCR

#### LCM-derived samples

Real time quantitative PCR was performed using Taqman probes (Life Technologies) and using the Applied Biosystems 7300 Real time PCR system (Life Technologies). The input for each assay was 50 ng of cDNA amplified from LCM-isolated RNA using the Ovation Pico WTA system V2 (NuGEN). Taqman Universal Master Mix II (Life Technologies) was used for all reactions. *C_t_* values were extracted from the fluorescence-versus-cycle measurements using the instrument software (Applied Biosystems). Relative mRNA levels were quantified from the *C_t_* values using the exp(ΔΔ*C_t_*) method [99], using *Gapdh* as the endogenous normalizer. The specific Taqman assays that were used for the study are listed in Table S13.

### Computational meta-analysis

Affymetrix microarray data from the Cho *et al.* study [100] and the Hägg *et al.* study [101] (see Table S5 for references) were obtained from NCBI GEO and processed using the Robust Multichip Average method [102] in the software package Bioconductor, to obtain background-adjusted, quantile-normalized, and probeset-summarized log_2_ intensities. Human gene probesets were mapped to mouse orthologs using the Bioconductor tools for querying NCBI HomoloGene, and differential expression testing was carried out using a two-way ANOVA with a Benjamini-Hochberg [103] false discovery rate cutoff of 0.15. From all other studies, lists of differentially expressed genes were obtained from data tables from the publications referenced in Table S5, and mapped to mouse orthologs using HomoloGene. For each mouse gene in the Entrez Gene database, the number of independent analyses in which the gene was reported as differentially expressed during foam cell formation or atherosclerosis progression (among the studies in Table S5) was tabulated and used as the gene's meta-analysis score. Genes with a score greater than or equal to two (see Table S2, column L) were counted as high-scoring in the meta-analysis, for statistical enrichment test (see Results). Statistical enrichment of the set of genes with meta-analysis scores of two or greater, within the set of 213 genes that are differentially expressed in plaque CD68+ cells, was tested using Fisher's Exact Test, with the larger sample set consisting of the set of 21,594 mouse genes for which a meta-analysis score was tabulated.

### Microarray data analysis

For microarray transcriptome profiling of plaque macrophages in the *Reversa* model, GeneChip scan images were processed into probe intensities using the Affymetrix GeneChip Operating Software system and then background-adjusted, quantile-normalized, and probeset-summarized using the Robust Multichip Average feature of Affymetrix Power Tools, with transcript-level probeset definitions from the Version 15 of the CustomCDF Project [104] (Ensembl Transcript-derived probeset definitions). Probe intensities were separately processed into exon-level normalized probeset intensities using the Robust Multichip Average Feature of Affymetrix Power Tools (version 1.10.2), and (separately) using exon-level probeset definitions from the CustomCDF project (Ensembl Exon) and from the Affymetrix Mouse Exon Array 1.0 ST NetAffx Annotations version 29. Exon-level detect-above-background calls were made using the two separate sets of (CustomCDF and Affymetrix) exon-level probesets using the Detection Above Background algorithm in Affymetrix Power Tools. For each exon-level probeset, detect-above-background *P* values were combined for all samples within a sample group using the geometric mean, and then across sample groups by taking the minimum *P* value, to obtain a single “present” *P* value for each exon-level probeset. For each Ensembl Transcript corresponding to one of the transcript-level probesets for which at least 75% of the constituent exon-level probesets was interrogated on the array, the transcript was considered to be above background in the sample if at least half of its constituent exons (that were interrogated with probes on the array) were detected above background with a “present” *P* value of less than or equal to 0.01 [105], [106]. For all probesets that passed the filter, log_2_ probeset intensities across all 24 *Reversa* samples (see Table S1) were analyzed for differential expression between the *Mttp*-inactivated and control sample groups, as described below.

For comparative transcriptome profiling of plaque macrophages in the transplant regression model, probe intensity values were obtained from a two-color 3′ microarray dataset of CD68+ cells from aortic arch grafts harvested from *Apoe* ^−/−^ and WT host animals three days post-surgery (*N* = 14 for *Apoe* ^−/−^, *N* = 18 for WT). This previously published dataset was obtained from the lab of one of the authors (EAF) [36], and was based on the Rosetta/Merck Mouse 23.6K 3.0 A1 microarray (custom-synthesized by Agilent; design files are available at GEO accession number GPL9733). Probe intensities in each of the two color channels (corresponding to sample-derived RNA and a universal mouse RNA reagent (Stratagene) that was used as an internal control) were combined to quantify absolute expression (geometric mean) and normalized expression (ratio of the macrophage sample-derived intensity to the internal control-derived intensity) for each of the samples. Probes were filtered for minimum absolute log_2_ intensity (greater than or equal to −5) to remove probable below-background probe intensities. For all probes that passed the filter, log_2_ expression ratios across all 32 samples were analyzed for differential expression between the WT and *Apoe* ^−/−^ sample groups, as described below.

Testing for differential expression was performed using a two-factor model (sex and treatment for the *Reversa* study, sex and genotype for the transplant study) with empirical Bayes variance estimates, using the Limma package in Bioconductor [107], with a proportion factor of 0.03. Transcripts were selected as differentially expressed between the sample groups if the treatment factor *P* value was less than 0.01. For each unique gene among the set of differentially expressed transcripts, a representative transcript was selected by selecting (from among the differentially expressed transcripts that are associated with the gene in the Ensembl database) the transcript with the largest number of exons represented on the microarray. Enrichment tests for gene annotation terms were carried out separately for the sets of upregulated and downregulated genes in the *Reversa* transcriptome dataset, using the gene annotation analysis tool DAVID [108] with a *P* value cutoff of 0.05. Gene sets were analyzed with three different functional annotation enrichment tools to determine whether gene annotations related to apoptosis or cell death are overrepresented: DAVID, Gene Set Enrichment Analysis [109], and Ingenuity Pathway Analysis (Ingenuity Systems, Redwood City, CA).

Microarray analysis of poly I:C-treated macrophages was carried out using the raw microarray intensity data from a previous study [110] in which primary murine bone marrow-derived macrophages (BMDM) were treated *in vitro* for two hours with 6 µg/mL poly I:C and then profiled using the Affymetrix Mouse Exon Array 1.0 ST GeneChip. Six CEL files (three from each of the two sample groups, untreated and poly I:C-treated) were obtained from NCBI GEO (accession number GSE7052) and processed as described above (save for the proportion factor used for the empirical Bayes step in LIMMA being 0.1). The statistical cutoff used for differential expression tests was *P*<0.01. The statistical test for overlap between the poly I:C-responsive genes in BMDM and the genes that are transcriptionally responsive to lipid lowering *in vivo* was carried out using Fisher's Exact Test (two-sided).

Microarray transcriptome profiling probe-level measurements from mouse peritoneal macrophages were quantile-normalized without background adjustment and then summarized for probeset intensities using the Affymetrix PLIER algorithm [111] and for detection above background using the Affymetrix DABG algorithm [112] using probe-to-probeset mappings from the CustomCDF project [104] version 15, based on the mouse transcripts and mouse exons lists from the Ensembl database release 65. Transcripts were marked as “present” only if at least 2/3 of the constituent exons had a DABG *P* value of less than 0.01 [105]. Differential expression testing for each Ensembl transcript was carried out using a *t*-test with empirical Bayes variance estimates, using the Limma package [107] in Bioconductor with 0.1 as the prior expected proportion of differentially expressed genes. *P* values for differential expression between the sample groups (vehicle and oxLDL) were converted to q-values using the Bioconductor package qvalue [113]. Transcripts were filtered with a false discovery rate cutoff of 0.05 and a minimum absolute fold-change cutoff of 2.0, and filtered to include only transcripts that could be mapped to the RefSeq NM identifier. Transcripts were mapped start site coordinates on the mm9 genome assembly, and noncoding sequences within ±5 kbp of the transcription start sites were retrieved, using the BioMart tool in Ensembl release 65.

### Transcription factor binding site motif analysis

In this section, we describe the computational method REMINISCE (Regulatory Element Machine-learning to Infer Networks Instructing Specific Cellular Expression; see Figure S1) that was used to identify transcription factor binding site motifs whose matches are overrepresented within 5′ regulatory regions of differentially expressed genes. Using the BioMart interface to the Ensembl database (release 65), transcription start site locations (on the NCBI M37 mouse genome assembly) were obtained for the sets of differentially expressed transcripts that were detected by microarray in CD68+ cells in plaques in the *Reversa* or transplant regression models. Noncoding DNA sequences (defined by excluding locations marked as protein coding within at least one exon within the Ensembl database) within ±5 kbp of a transcription start site were analyzed using a Random Forest classifier [114] to identify probable *cis*-regulatory regions, with a score assigned at 100 bp intervals based on the voting fraction of trees in the classifier. The features used for the classification (Table S8) include genome-wide measurements (binarized peak locations) of histone acetylation in bone marrow-derived macrophages obtained by ChIP-seq [39], “valley scores” in histone acetylation ChIP-seq signal [39], DNase I hypersensitive sites in bone marrow-derived macrophages [39] detected by short-read sequencing, vertebrate PhastCons interspecies sequence conservation scores from the University of California Santa Cruz Genome Bioinformatics database [115], GC content, computational predictions of CpG island locations [116], and basepair distances to the nearest binarized feature locations of each type. The Random Forest classifier for predicting *cis*-regulatory regions was trained using compendium of transcription factor binding site locations obtained by combining ChIP-seq datasets targeting 19 transcriptional regulators in murine macrophages, that were obtained from the literature (Table S8). Noncoding sequence within regions predicted to have a *cis*-regulatory function by the Random Forest model (voting fraction greater than 0.5) was scanned for transcription factor binding site motif matches using a combined set of vertebrate binding site position weight matrices from the TRANSFAC Professional 2010 [117] and JASPAR 2010 [118] databases. The scanning was performed using the software tool Clover [119] (Feb. 19, 2010 release) on noncoding sequences within *cis*-regulatory regions (after masking low-complexity repeats using NCBI DustMasker) proximal to transcription start sites of differentially expressed transcripts as well as proximal to transcription start sites of a set of 1,000 randomly selected genes that were detected above background in plaque macrophages by microarray hybridization (see Microarray Analysis). For the Clover scanning, a pseudocount value of 0.3 [120] was used. Matches for binding site sequence patterns within the promoter sequences for upregulated or downregulated genes were assessed for statistical overrepresentation using the frequencies compiled on the promoter regions for the 1,000 randomly selected macrophage-expressed genes, using the binomial test (*P* value threshold of 0.01). Subsequent to the motif analysis using TRANSFAC 2010, 5′ *cis*-regulatory regions for the upregulated *Reversa* gene set were re-analyzed using a substantially larger set (numbering 56) of TCF/LEF transcription factor family motifs from an updated motif database (TRANSFAC Professional 2013.4), using the same methods.

### Transcription factor network analysis

Official gene symbols for genes encoding protein components of transcription factors that were implicated in the transcription factor binding site enrichment analysis (see Figure 3A) were uploaded to the GeneMANIA network analysis web application [59]. The interaction network for the transcription factors was assembled using protein-protein interactions (direct physical interactions only), and visualized using Cytoscape [121].

### MicroRNA target sequence enrichment analysis

MicroRNA enrichment analysis was carried out using the CORNA software algorithm [122] using conserved microRNA target predictions from miRBase (v5) and using Ensembl BioMart [123] (release 70) for gene identifier translation. Statistical enrichment was assessed using Fisher's Exact Test (*P* value threshold of 0.01). Pathway enrichment analysis of genes with target sequences for the microRNAs shown in Figure S4 was carried out using the analysis program DIANA-miRPath [65], with target sequences predictions from the DIANA-microT-CDS v.5.0 algorithm [124].

### Human SNP analysis of plaque regression gene sets

A set of 239,165 eSNP-to-gene associations (that collectively connect 157,668 eSNPs to monocyte expression levels of 13,989 genes) was compiled by merging (eSNP,gene) pair lists from two human population genetic studies of monocyte gene expression regulation [48], [49] comprised of samples from 1,490 and 283 individuals, respectively. From these 239,165 pairs, eSNPs that were associated with human genes whose mouse orthologs (obtained via successive mapping using the HomoloGene version 68 and the INPARANOID [125] version 8.0 databases) were detected as differentially expressed in plaque CD68+ cells in the *Reversa* model of plaque regression (for *Reversa*, the 213 genes in Table S2) or in the aortic transplant model of plaque regression (from Table S2 of [36], the 549 genes with absolute geometric-mean expression ratio >1.25 between the WT and *Apoe* ^−/−^ recipient sample groups) were then obtained, resulting in lists of 2,380 (*Reversa*) and 5,024 (transplant) plaque regression-associated eSNPs, respectively. The three sets of eSNPs – *Reversa* eSNPs, transplant eSNPs, and the full 157,668 “monocyte eSNPs” – were expanded to include proxy SNPs that are both in linkage disequilibrium (LD) with an eSNP (*R*>0.9) and within 250 kbp of the eSNP, yielding LD-expanded sets of 6,214, 13,248, and 363,283 SNPs, respectively. The expansion to proxy SNPs was carried out using LD maps from the 1,000 Genomes Project (CEU population group) and using the SNP analysis software SNAP [126]. A set of 761 SNPs that are associated with risk of atherosclerosis or CAD was obtained by searching the NCBI Phenotype-Genotype Integrator database [51] for the MeSH terms “atherosclerosis”, “coronary artery disease”, and “carotid stenosis”. Overlaps were computed for the set of 761 atherosclerosis/CAD SNPs with four different sets of SNPs: the 6,214 LD-expanded eSNPs from the *Reversa* model (overlap = 6), the 13,248 LD-expanded eSNPs from the aortic transplant model (overlap = 11), the 363,283 LD-expanded monocyte eSNPs (overlap = 134), and the set of all 44,278,189 validated SNPs in dbSNP build 138 (overlap = 761). From these overlaps, two 2×2 contingency tables were constructed to test for association between atherosclerosis/CAD SNPs and regression gene-associated eSNPs in the *Reversa* and aortic transplant models, respectively, using the LD-expanded set of monocyte eSNPs as a background set. Additionally, two 2×2 contingency tables were constructed to test for association between atherosclerosis/CAD SNPs and regression gene-associated eSNPs in the *Reversa* and aortic transplant models, respectively, using the set of all validated SNPs as a background set. *P* values and odds ratios (Figure 2C and Table S6) were obtained for these four contingency tables using Fisher's Exact Test. Confidence intervals (shown in Figure 2C) on the expected overlap of atherosclerosis/CAD SNPs and regression-associated eSNPs were obtained using the quantile function of the hypergeometric distribution.

### Analysis software

Computational analyses were carried using scripts in the R statistical computing environment, also using the Bioconductor set of packages for R), and in the Perl programming language. Bar graphs were prepared using Prism (GraphPad Software, La Jolla, CA). All custom software scripts used in the computational analysis are available from the authors upon request. Statistical tests were carried out using R and GraphPad Prism.

## Supporting Information

Figure S1 Diagram of REMINISCE method for detecting enrichments of transcription factor binding sites for specific transcription factors, within predicted regulatory regions. Cell type-specific epigenomic and chromatin information (A) are combined with cell type-generic genomic information (B) within an ensemble decision tree classifier, Random Forest (C). The classifier is trained to use the input features (A,B) to predict the locations of *cis*-regulatory regions, identified by combining transcription factor location datasets (D) from the specific cell type (macrophages). The classifier predicts *cis*-regulatory regions (E) upstream of genes that are differentially expressed in plaque macrophages in response to *lipid lowering in vivo*, and these regions are scanned to identify matches for transcription factor binding site motifs from a precompiled library of vertebrate motifs (F). For each transcription factor, frequencies per bp of predicted *cis*-regulatory region sequence for its binding sites are tabulated and tested for enrichment (G) above a background frequency from *cis*-regulatory regions for a list of genes that are expressed above background level in murine macrophages. See Methods section for details.
(TIFF)

Figure S2 Sensitivity for *in silico* detection of enrichments of transcription factor binding sites is compared between the epigenome-guided method, REMINISCE, and approaches in which all noncoding sequence within ±1 kbp or ±5 kbp of transcription start sites are analyzed. The three analysis methods were applied to a transcriptome profiling study of macrophage foam cells in which mouse resident peritoneal macrophages (*N* = 3 independent replicate experiments; see Methods) were cultured with oxLDL or vehicle for 24 h. High-confidence sets of differentially expressed genes were identified (false discovery rate cutoff of 0.05 and minimum absolute fold-change of 2.0) as upregulated (94 genes) or downregulated (342 genes), and a background set of 2,000 genes that were randomly selected from the set of genes that were detected above background in at least one sample group (vehicle or oxLDL). The 5′ regulatory regions for the genes were scanned for matches to transcription factor binding site motifs using the epigenome-guided method, REMINISCE, as described in the Methods section (“REMINISCE method”, white bars) and by analyzing all noncoding sequence within ±1 kbp of transcription start sites (“1 kbp classic”, gray bars) or within ±5 kbp of transcription start sites (“5 kbp classic”, charcoal bars). For all three methods, the number of matches for a binding site motif in the biological gene sets (genes up- or downregulated by oxLDL) per kbp of sequence analyzed, was compared to the number that would be expected by chance for genes that are expressed above background in resident peritoneal macrophages. Bar group labels represent transcription factor binding site motifs. Bar lengths represent the ratio of the number of motif matches (per kbp of sequence analyzed) for the indicated oxLDL-response gene set (left, upregulated in oxLDL; right, downregulated in oxLDL) to the number of motif matches (per kbp of sequence analyzed) for the background set of genes. For each bar group (motif), a missing bar of a particular shade indicates that the motif's binding sites were not detected as enriched (*P*<0.01) using that method. The results indicate that the REMINISCE method detected substantially more motifs, and at higher enrichment ratios, than the two methods based on analysis of all noncoding sequence.
(TIFF)

Figure S3 Motif scanning enrichment analysis for promoters of genes that are differentially expressed in plaque macrophages during plaque regression, in the Reversa and/or in the transplant regression models. Bars represent the ratio of the number of transcription factor (TF) binding site motif matches (per kbp of *cis*-regulatory sequence analyzed) for the indicated differentially expressed gene set, to the number of binding site motif matches per kbp for randomly generated sets of genes that are expressed above background in plaque CD68+ cells. Bar shading indicates the regression model from which the gene set was derived (white, *Reversa* model, gray, transplant regression model). A missing gray bar indicates that binding sites for the indicated TF binding site motif were not detected as significantly higher than the number expected by chance, for the transplant regression model. TF motifs shown on this figure were selected based on a significance threshold (see Methods) for enrichment for gene sets derived from the *Reversa* mouse, and thus, every TF shown here has a white bar. (A) TF binding site motifs for genes that are upregulated in CD68+ cells from regressing animals vs. control animals. (B) TF binding site motifs for genes that are downregulated in CD68+ cells from regressing animals vs. control animals.
(TIFF)

Figure S4 microRNA target sequence enrichment analysis identifies many microRNAs whose target sequences are overrepresented within the 3′ UTRs of genes that are differentially expressed in CD68+ cells in plaques in *Mttp*-inactivated vs. vehicle-treated *Reversa* mice. (A) Enrichment analysis for genes that are upregulated in *Mttp*-inactivated vs. vehicle-treated macrophages. Bars indicate the ratio of the number of target sequence matches within the gene set, vs. the number expected by chance for randomly selected sets of genes (see Methods). (B) Enrichment analysis for genes that are downregulated in *Mttp*-inactivated vs. vehicle-treated macrophages.
(TIFF)

Figure S5 Upregulation of Wnt pathway target genes in CD68+ cells in regressing vs. progressing plaques. qPCR-measured relative mRNA levels for Wnt signaling pathway target genes *Lrp6* (A) and *Gja1* (B) in CD68+ cells in aortic grafts in *Apoe* ^−/−^ and WT recipient animals (*N* = 6 per genotype group) in the aortic transplant regression model on day five post-transplant show increased expression during plaque regression vs. progression. Bars, mean ± SE. *P* values reported are for an unpaired, two-tailed Student's *t*-test.
(TIFF)

Figure S6 Canonical Wnt pathway activation stimulates macrophage migration *in vitro*. (A) Micrographs of a scratch-wound migration assay for primary murine macrophages incubated for 24 h in medium alone or medium plus Wnt3a (400 ng/mL). Dotted black lines demarcate the scratch region. (B) Quantification of percentage of scratch area recovered after 24 h incubation. Bars, mean ± SE (*N* = 4 replicates per sample group). (‡) *P*<0.01 (unpaired, two-tailed Student's *t*-test vs. control).
(TIFF)

Figure S7 β-catenin immunofluorescence has minimal background signal in the arterial intima of aortic graft sections from the aortic transplant model of plaque regression. Representative images of the negative control (primary antibody excluded) are shown, with and without DAPI merge. The elastic lamina is identified by its autofluorescence. Green, β-catenin immunofluorescence; blue, DAPI fluorescence. Scale bar, 100 µm.
(TIFF)

Figure S8 β-catenin immunofluorescence shows increased abundance in the nuclear area, in WT vs. Apoe^−/−^ recipient mice in the aortic transplant regression model. (A) Images are confocal fluorescence micrographs from representative sections from a total of six grafts (*N* = 3 for each recipient animal genotype; six sections per graft). Green, β-catenin immunofluorescence; blue, DAPI fluorescence; dashed white line divides lumen from intima; scale bars, 20 µm. White arrows indicate areas of overlap of β-catenin and DAPI fluorescence signals in the regressing plaque section (bottom image). (B) Zoomed images of regions that are indicated with yellow dotted lines in the merged images from progressing and regressing plaques (A, second and fourth images from the top).
(TIFF)

Table S1 List of sample groups for the transcriptome profiling of plaque macrophages from the Reversa plaque regression model. Columns are (A) genotype; (B) sex; (C) treatment; (D) time point (from last injection of poly I:C for the poly I:C treatment group); (E) number of animals in the sample group.
(XLSX)

Table S2 Genes that were detected as differentially expressed in plaque macrophages from *Mttp*-inactivated vs. vehicle-treated animals, in the *Reversa* model. Columns are: (A) Ensembl Gene ID; (B) Chromosome; (C) Strand; (D) Transcript start coordinate; (E) Transcript end coordinate; (F) Official gene symbol; (G) Gene description; (H) RefSeq ID (if applicable); (I) Entrez Gene ID(s); (J) *P* value for the treatment factor (poly I:C vs. saline) in the statistical analysis; (K) signed ratio of the average expression levels between the sample groups (*Mttp*-inactivated and vehicle-treated groups); (L) meta-analysis score (see section Meta Analysis, in Methods); an empty cell indicates a lack of gene symbol mapping from the meta-analysis).
(XLSX)

Table S3 Genes that are differentially expressed by poly I:C treatment in bone marrow-derived macrophages. Columns are: (A) Ensembl Transcript ID; (B) Chromosome; (C) Strand; (D) Transcript start coordinate; (E) Transcript end coordinate; (F) Official gene symbol; (G) Gene description; (H) RefSeq ID (if applicable); (I) Entrez Gene ID(s); (J) Entrez Gene ID(s); (K) signed ratio of the average expression levels between the sample groups (poly I:C and unstimulated, respectively).
(XLSX)

Table S4 Gene functional enrichment analysis results for genes that are differentially expressed in plaque CD68+ cells in *Mttp*-inactivated vs. vehicle-treated *Reversa* mice. Columns are: (A) Type of gene function annotation; (B) Gene function term; (C) Count of genes with this term; (D) Percent of genes with this term; (E) *P* value for enrichment (Fisher's Exact Test); (F) Ensembl Gene IDs for genes with this term.
(XLSX)

Table S5 Studies from the literature that were used in the meta-analysis of gene expression changes during foam cell formation and atherosclerosis. Columns are (A) Authors; (B) Journal; (C) Bibliographic reference; (D) Organism; (E) Cell/tissue type; (F) treatment, and (G) study type.
(XLSX)

Table S6 Numbers of risk SNPs for atherosclerosis or CAD that coincide with monocyte expression QTLs and with regression-associated monocyte expression QTLs. (see Figure 2C and Methods). Columns are as follows: (A) Row labels; (B) Starting total SNP counts; (C) Number of SNPs after expanding to proxy SNPs within an LD block; (D) Number that overlap with known risk SNPs for atherosclerosis or CAD; (E) Frequency of atherosclerosis/CAD SNPs within each row's LD-expanded SNP set; (F) *P* value for enrichment of atherosclerosis/CAD SNPs (Fisher's Exact Test); (G) Odds ratio from Fisher's Exact Test.
(XLSX)

Table S7 Risk SNPs for atherosclerosis or CAD that coincide with regression-associated monocyte expression QTLs. Columns are as follows: (A) RefSNP ID; (B) Trait (MeSH term); (C) Context (location relative to nearest gene; “nearGene-3” indicates that the SNP is located downstream of the 3′ end of a gene).
(XLSX)

Table S8 Sources of epigenomic and transcription factor binding site ChIP-seq data used for features and training (respectively) for the computational model for mapping *cis*-regulatory regions Columns are as follows: (A) Transcriptional regulator or chromatin mark; (B) Proposed function; (C) Source; (D) Accession number of the ChIP-seq data set (where applicable).
(XLSX)

Table S9 Statistically overrepresented motifs within up- or down-regulated genes in the *Reversa* model. Results for the sets of upregulated and downregulated genes are presented in separate blocks of rows, each labeled “for upregulated genes” and “for downregulated genes” respectively. The results of a motif scan of the upregulated genes using TCF/LEF family motifs from TRANSFAC 2013 are reported in a third block of rows, marked “TCF/LEF Scan of upregulated genes with TRANSFAC 2013”. Columns are as follows: (A) Motif ID; (B) Motif description (note: “HS” indicates a half-site for a bipartite binding site sequence); (C) Consensus sequence pattern; (D) Binding site match frequency enrichment ratio in the differentially expressed gene set vs. the frequency expected by chance among macrophage-expressed genes; (E) *P* value for the binomial test on the frequency of matches; binding site frequency enrichment ratio in differentially expressed genes in the transplant regression model.
(XLSX)

Table S10 Numbers of genes with transcription factor binding site sequence matches of the indicated types, within their promoter regions. Results for the sets of upregulated and downregulated genes are presented in separate sections labeled “for upregulated genes” and “for downregulated genes” respectively. Columns are: (A) motif identifier; (B) motif description; (C) number of genes possessing a match for the motif within their promoter regions; (D) fraction of genes; (E) number of cytoskeleton-related genes (based on GO annotation) possessing a match for the motif within their promoter regions.
(XLSX)

Table S11 Results of microRNA target sequence enrichment analysis for sets of genes that are differentially expressed in plaque CD68+ cells from *Mttp*-inactivated vs. vehicle-treated *Reversa* mice. Columns are as follows: (A) MicroRNA name; (B) Total number of target sequences among all mouse genes; (C) Expected number of target sequences for the indicated gene set (upregulated or downregulated genes); (D) Observed number of target sequences for the indicated gene set; (E) *P* value from the hypergeometric test; (F) ratio of observed to expected count.
(XLSX)

Table S12 Results of pathway enrichment analysis of genes possessing target sequences for microRNAs listed in Figure 4A, derived from transcriptome profiling of plaque CD68+ cells from *Reversa* mice in plaque regression. Columns are as follows: (A) Pathway/function name; (B) Pathway ID; (C) Number of genes in the pathway that have a target sequence for one of the microRNAs in Figure S4A; (D) *P* value for enrichment test; (E) Official gene symbols; (F) Ensembl Gene IDs.
(XLSX)

Table S13 qPCR primers that were used in this study. Columns are: (A) Official gene symbol; (B) Taqman assay ID.
(XLSX)

## References

[1] Alwan A, Armstrong T, Bettcher D, Boerma T, Branca F, et al. (2011) Global Atlas on Cardiovascular Diseases Prevention and Control. Mendes S, Puska P, Norrving B, editors Geneva: World Health Organization.

[2] Oliva A, Flores J, Merigioli S, LeDuc L, Benito B, et al (2011) Autopsy investigation and Bayesian approach to coronary artery disease in victims of motor-vehicle accidents. Atherosclerosis 218: 28–32 10.1016/j.atherosclerosis.2011.05.012 21663913

[3] McGill HC, McMahan CA, Gidding SS (2008) Preventing heart disease in the 21st century: implications of the Pathobiological Determinants of Atherosclerosis in Youth (PDAY) study. Circulation 117: 1216–1227 10.1161/CIRCULATIONAHA.107.717033 18316498

[4] Wilson PW, D'Agostino RB, Levy D, Belanger AM, Silbershatz H, et al (1998) Prediction of coronary heart disease using risk factor categories. Circulation 97: 1837–1847 10.1161/01.CIR.97.18.1837 9603539

[5] Reardon MF, Nestel PJ, Craig IH, Harper RW (1985) Lipoprotein predictors of the severity of coronary artery disease in men and women. Circulation 71: 881–888 10.1161/01.CIR.71.5.881 3986978

[6] Williams KJ, Feig JE, Fisher EA (2008) Rapid regression of atherosclerosis: insights from the clinical and experimental literature. Nat Clin Pract Cardiovasc Med 5: 91–102 10.1038/ncpcardio1086 18223541

[7] Francis AA, Pierce GN (2011) An integrated approach for the mechanisms responsible for atherosclerotic plaque regression. Exp Clin Cardiol 16: 77–86. 22065938 PMC3209544

[8] Hewing B, Fisher EA (2012) Preclinical mouse models and methods for the discovery of the causes and treatments of atherosclerosis. Expert Opin Drug Discov 7: 207–216 10.1517/17460441.2012.660143 22468952 PMC3612348

[9] Hansson GK, Hermansson A (2011) The immune system in atherosclerosis. Nat Immunol 12: 204–212 10.1038/ni.2001 21321594

[10] Moore KJ, Tabas I (2011) Macrophages in the pathogenesis of atherosclerosis. Cell 145: 341–355. 21529710 10.1016/j.cell.2011.04.005 PMC3111065

[11] Li AC, Glass CK (2002) The macrophage foam cell as a target for therapeutic intervention. Nat Med 8: 1235–1242 10.1038/nm1102-1235 12411950

[12] Ross R (1999) Atherosclerosis–an inflammatory disease. N Engl J Med 340: 115–126 10.1056/NEJM199901143400207 9887164

[13] Shah PK, Falk E, Badimon JJ, Fernandez-Ortiz A, Mailhac A, et al (1995) Human monocyte-derived macrophages induce collagen breakdown in fibrous caps of atherosclerotic plaques. Potential role of matrix-degrading metalloproteinases and implications for plaque rupture. Circulation 92: 1565–1569. 7664441

[14] Libby P, Geng YJ, Aikawa M, Schoenbeck U, Mach F, et al (1996) Macrophages and atherosclerotic plaque stability. Curr Opin Lipidol 7: 330–335. 8937525 10.1097/00041433-199610000-00012

[15] Daoud AS, Fritz KE, Jarmolych J, Frank AS (1985) Role of macrophages in regression of atherosclerosis. Ann N Y Acad Sci 454: 101–114. 3865603 10.1111/j.1749-6632.1985.tb11848.x

[16] Tangirala RK, Tsukamoto K, Chun SH, Usher D, Pure E, et al (1999) Regression of atherosclerosis induced by liver-directed gene transfer of apolipoprotein A-I in mice. Circulation 100: 1816–1822 10.1161/01.CIR.100.17.1816 10534470

[17] Potteaux S, Gautier EL, Hutchison SB, van Rooijen N, Rader DJ, et al (2011) Suppressed monocyte recruitment drives macrophage removal from atherosclerotic plaques of *Apoe* ^−/−^ mice during disease regression. J Clin Invest 121: 2025–2036 10.1172/JCI43802 21505265 PMC3083793

[18] Raffai RL, Weisgraber KH (2002) Hypomorphic apolipoprotein E mice: a new model of conditional gene repair to examine apolipoprotein E-mediated metabolism. J Biochem 277: 11064–11068 10.1074/jbc.M111222200 11792702

[19] Lieu HD, Withycombe SK, Walker Q, Rong JX, Walzem RL, et al (2003) Eliminating atherogenesis in mice by switching off hepatic lipoprotein secretion. Circulation 107: 1315–1321. 12628954 10.1161/01.cir.0000054781.50889.0c

[20] Feig JE, Parathath S, Rong JX, Mick SL, Vengrenyuk Y, et al (2011) Reversal of hyperlipidemia with a genetic switch favorably affects the content and inflammatory state of macrophages in atherosclerotic plaques. Circulation 123: 989–998 10.1161/CIRCULATIONAHA.110.984146 21339485 PMC3131163

[21] Trogan E, Fayad ZA, Itskovich VV, Aguinaldo J-GS, Mani V, et al (2004) Serial studies of mouse atherosclerosis by *in vivo* magnetic resonance imaging detect lesion regression after correction of dyslipidemia. Arterioscler Thromb Vasc Biol 24: 1714–1719 10.1161/01.ATV.0000139313.69015.1c 15256400

[22] Llodra J, Angeli V, Liu J, Trogan E, Fisher EA, et al (2004) Emigration of monocyte-derived cells from atherosclerotic lesions characterizes regressive, but not progressive, plaques. Proc Natl Acad Sci USA 101: 11779–11784 10.1073/pnas.0403259101 15280540 PMC511052

[23] Trogan E, Feig JE, Dogan S, Rothblat GH, Angeli V, et al (2006) Gene expression changes in foam cells and the role of chemokine receptor CCR7 during atherosclerosis regression in ApoE-deficient mice. Proc Natl Acad Sci U S A 103: 3781–3786 10.1073/pnas.0511043103 16537455 PMC1450154

[24] Feig JE, Shang Y, Rotllan N, Vengrenyuk Y, Wu C, et al (2011) Statins promote the regression of atherosclerosis via activation of the CCR7-dependent emigration pathway in macrophages. PLoS ONE 6: e28534 10.1371/journal.pone.0028534 22163030 PMC3232231

[25] Toomey S, Harhen B, Roche HM, Fitzgerald D, Belton O (2006) Profound resolution of early atherosclerosis with conjugated linoleic acid. Atherosclerosis 187: 40–49 10.1016/j.atherosclerosis.2005.08.024 16182300

[26] Hewing B, Parathath S, Mai CK, Fiel MI, Guo L, et al (2013) Rapid regression of atherosclerosis with MTP inhibitor treatment. Atherosclerosis 227: 125–129 10.1016/j.atherosclerosis.2012.12.026 23332773 PMC4047651

[27] Shah PK, Yano J, Reyes O, Chyu KY, Kaul S, et al (2001) High-dose recombinant apolipoprotein A-I_Milano_ mobilizes tissue cholesterol and rapidly reduces plaque lipid and macrophage content in apolipoprotein E-deficient mice. Potential implications for acute plaque stabilization. Circulation 103: 3047–3050 10.1161/hc2501.092494 11425766

[28] Feig JE, Rong JX, Shamir R, Sanson M, Vengrenyuk Y, et al (2011) HDL promotes rapid atherosclerosis regression in mice and alters inflammatory properties of plaque monocyte-derived cells. Proceedings of the National Academy of Sciences 108: 7166–7171 10.1073/pnas.1016086108 PMC3084076 21482781

[29] Skogsberg J, Lundstrom J, Kovacs A, Nilsson R, Noori P, et al (2008) Transcriptional profiling uncovers a network of cholesterol-responsive atherosclerosis target genes. PLoS Genet 4: e1000036 10.1371/journal.pgen.1000036 18369455 PMC2265530

[30] Björkegren JLM, Hägg S, Talukdar HA, Foroughi Asl H, Jain RK, et al (2014) Plasma cholesterol–induced lesion networks activated before regression of early, mature, and advanced atherosclerosis. PLoS Genet 10: e1004201 10.1371/journal.pgen.1004201.s019 24586211 PMC3937269

[31] Chereshnev I, Trogan E, Omerhodzic S, Itskovich V, Aguinaldo J-G, et al (2003) Mouse model of heterotopic aortic arch transplantation. J Surg Res 111: 171–176 10.1016/S0022-4804(03)00039-8 12850459

[32] Véniant MM, Zlot CH, Walzem RL, Pierotti V, Driscoll R, et al (1998) Lipoprotein clearance mechanisms in LDL receptor-deficient “Apo-B48-only” and “Apo-B100-only” mice. J Clin Invest 102: 1559–1568 10.1172/JCI4164 9788969 PMC509006

[33] Véniant MM, Sullivan MA, Kim SK, Ambroziak P, Chu A, et al (2000) Defining the atherogenicity of large and small lipoproteins containing apolipoprotein B100. J Clin Invest 106: 1501–1510 10.1172/JCI10695 11120757 PMC387257

[34] Raabe M, Véniant MM, Sullivan MA, Zlot CH, Bjorkegren J, et al (1999) Analysis of the role of microsomal triglyceride transfer protein in the liver of tissue-specific knockout mice. J Clin Invest 103: 1287–1298 10.1172/JCI6576 10225972 PMC408359

[35] Plump AS, Smith JD, Hayek T, Aalto-Setälä K, Walsh A, et al (1992) Severe hypercholesterolemia and atherosclerosis in apolipoprotein E-deficient mice created by homologous recombination in ES cells. Cell 71: 343–353. 1423598 10.1016/0092-8674(92)90362-g

[36] Feig JE, Vengrenyuk Y, Reiser V, Wu C, Statnikov A, et al (2012) Regression of atherosclerosis is characterized by broad changes in the plaque macrophage transcriptome. PLoS ONE 7: e39790 10.1371/journal.pone.0039790.t001 22761902 PMC3384622

[37] Martinez FO, Gordon S, Locati M, Mantovani A (2006) Transcriptional profiling of the human monocyte-to-macrophage differentiation and polarization: new molecules and patterns of gene expression. J Immunol 177: 7303–7311. 17082649 10.4049/jimmunol.177.10.7303

[38] Vettese-Dadey M, Grant PA, Hebbes TR, Crane- Robinson C, Allis CD, et al (1996) Acetylation of histone H4 plays a primary role in enhancing transcription factor binding to nucleosomal DNA *in vitro* . EMBO J 15: 2508–2518. 8665858 PMC450183

[39] Ramsey SA, Knijnenburg TA, Kennedy KA, Zak DE, Gilchrist M, et al (2010) Genome-wide histone acetylation data improve prediction of mammalian transcription factor binding sites. Bioinformatics 26: 2071–2075 10.1093/bioinformatics/btq405 20663846 PMC2922897

[40] Song L, Zhang Z, Grasfeder LL, Boyle AP, Giresi PG, et al (2011) Open chromatin defined by DNaseI and FAIRE identifies regulatory elements that shape cell-type identity. Genome Res 21: 1757–1767 10.1101/gr.121541.111 21750106 PMC3202292

[41] Logan CY, Nusse R (2004) The Wnt signaling pathway in development and disease. Annu Rev Cell Dev Biol 20: 781–810 10.1146/annurev.cellbio.20.010403.113126 15473860

[42] Mosimann C, Hausmann G, Basler K (2009) Beta-catenin hits chromatin: regulation of Wnt target gene activation. Nat Rev Mol Cell Biol 10: 276–286 10.1038/nrm2654 19305417

[43] Pereira CP, Bachli EB, Schoedon G (2009) The Wnt pathway: a macrophage effector molecule that triggers inflammation. Curr Atheroscler Rep 11: 236–242. 19361356 10.1007/s11883-009-0036-4

[44] Amini-Nik S, Cambridge E, Yu W, Guo A, Whetstone H, et al (2014) β-Catenin-regulated myeloid cell adhesion and migration determine wound healing. J Clin Invest 124: 2599–2610 10.1172/JCI62059 24837430 PMC4089463

[45] Trogan E, Choudhury RP, Dansky HM, Rong JX, Breslow JL, et al (2002) Laser capture microdissection analysis of gene expression in macrophages from atherosclerotic lesions of apolipoprotein E-deficient mice. Proc Natl Acad Sci U S A 99: 2234–2239 10.1073/pnas.042683999 11842210 PMC122348

[46] Trogan E, Fisher EA (2005) Laser capture microdissection for analysis of macrophage gene expression from atherosclerotic lesions. Methods Mol Biol 293: 221–231. 16028422 10.1385/1-59259-853-6:221

[47] English SB, Butte AJ (2007) Evaluation and integration of 49 genome-wide experiments and the prediction of previously unknown obesity-related genes. Bioinformatics 23: 2910–2917 10.1093/bioinformatics/btm483 17921495 PMC2839901

[48] Zeller T, Wild P, Szymczak S, Rotival M, Schillert A, et al (2010) Genetics and beyond–the transcriptome of human monocytes and disease susceptibility. PLoS ONE 5: e10693 10.1371/journal.pone.0010693.t007 20502693 PMC2872668

[49] Fairfax BP, Makino S, Radhakrishnan J, Plant K, Leslie S, et al (2012) Genetics of gene expression in primary immune cells identifies cell type-specific master regulators and roles of HLA alleles. Nat Genet 44: 502–510 10.1038/ng.2205 22446964 PMC3437404

[50] Genomes Project Consortium (2012) Abecasis GR, Auton A, Brooks LD, DePristo MA, et al (2012) An integrated map of genetic variation from 1,092 human genomes. Nature 491: 56–65 10.1038/nature11632 23128226 PMC3498066

[51] Ramos EM, Hoffman D, Junkins HA, Maglott D, Phan L, et al (2014) Phenotype-Genotype Integrator (PheGenI): synthesizing genome-wide association study (GWAS) data with existing genomic resources. Eur J Hum Genet 22: 144–147 10.1038/ejhg.2013.96 23695286 PMC3865418

[52] Kleber ME, Renner W, Grammer TB, Linsel-Nitschke P, Boehm BO, et al (2010) Association of the single nucleotide polymorphism rs599839 in the vicinity of the sortilin 1 gene with LDL and triglyceride metabolism, coronary heart disease and myocardial infarction. The Ludwigshafen Risk and Cardiovascular Health Study. Atherosclerosis 209: 492–497 10.1016/j.atherosclerosis.2009.09.068 19837406

[53] Zhou L, Ding H, Zhang X, He M, Huang S, et al (2011) Genetic variants at newly identified lipid loci are associated with coronary heart disease in a Chinese Han population. PLoS ONE 6: e27481 10.1371/journal.pone.0027481 22110658 PMC3215720

[54] Samani NJ, Erdmann J, Hall AS, Hengstenberg C, Mangino M, et al (2007) Genomewide association analysis of coronary artery disease. N Engl J Med 357: 443–453 10.1056/NEJMoa072366 17634449 PMC2719290

[55] Coronary Artery Disease Consortium (2009) Samani NJ, Deloukas P, Erdmann J, Hengstenberg C, et al (2009) Large scale association analysis of novel genetic loci for coronary artery disease. Arterioscler Thromb Vasc Biol 29: 774–780 10.1161/ATVBAHA.108.181388 19164808 PMC3315048

[56] Musunuru K, Strong A, Frank-Kamenetsky M, Lee NE, Ahfeldt T, et al (2010) From noncoding variant to phenotype via SORT1 at the 1p13 cholesterol locus. Nature 466: 714–719 10.1038/nature09266 20686566 PMC3062476

[57] Kjolby M, Andersen OM, Breiderhoff T, Fjorback AW, Pedersen KM, et al (2010) Sort1, encoded by the cardiovascular risk locus 1p13.3, is a regulator of hepatic lipoprotein export. Cell Metab 12: 213–223 10.1016/j.cmet.2010.08.006 20816088

[58] Shen Y, Yue F, McCleary DF, Ye Z, Edsall L, et al (2012) A map of the *cis*-regulatory sequences in the mouse genome. Nature 488: 116–120 10.1038/nature11243 22763441 PMC4041622

[59] Mostafavi S, Ray D, Warde-Farley D, Grouios C, Morris Q (2008) GeneMANIA: a real-time multiple association network integration algorithm for predicting gene function. Genome Biol 9 Suppl 1: S4 10.1186/gb-2008-9-s1-s4 PMC2447538 18613948

[60] Stumpf M, Balding DJ, Girolami M (2011) Handbook of Statistical Systems Biology. First ed. West Sussex: John W. Wiley & Sons.

[61] Gabdoulline R, Eckweiler D, Kel A, Stegmaier P (2012) 3DTF: a web server for predicting transcription factor PWMs using 3D structure-based energy calculations. Nucleic Acids Research 40: W180–W185 10.1093/nar/gks551 22693215 PMC3394331

[62] Tsaousi A, Mill C, George SJ (2011) The Wnt pathways in vascular disease: lessons from vascular development. Curr Opin Lipidol 22: 350–357 10.1097/MOL.0b013e32834aa701 21841485

[63] Mermelstein CS, Portilho DM, Mendes FA, Costa ML, Abreu JG (2007) Wnt/beta-catenin pathway activation and myogenic differentiation are induced by cholesterol depletion. Differentiation 75: 184–192 10.1111/j.1432-0436.2006.00129.x 17359297

[64] Kormish JD, Sinner D, Zorn AM (2010) Interactions between SOX factors and Wnt/beta-catenin signaling in development and disease. Dev Dyn 239: 56–68 10.1002/dvdy.22046 19655378 PMC3269784

[65] Vlachos IS, Kostoulas N, Vergoulis T, Georgakilas G, Reczko M, et al (2012) DIANA miRPath v.2.0: investigating the combinatorial effect of microRNAs in pathways. Nucleic Acids Research 40: W498–W504 10.1093/nar/gks494 22649059 PMC3394305

[66] Mani A, Radhakrishnan J, Wang H, Mani A, Mani M-A, et al (2007) *LRP6* mutation in a family with early coronary disease and metabolic risk factors. Science 315: 1278–1282 10.1126/science.1136370 17332414 PMC2945222

[67] van der Heyden MA, Rook MB, Hermans MM, Rijksen G, Boonstra J, et al (1998) Identification of connexin43 as a functional target for Wnt signalling. J Cell Sci 111 (Pt 12) 1741–1749. 9601103 10.1242/jcs.111.12.1741

[68] Todaro GJ, Lazar GK, Green H (1965) The initiation of cell division in a contact-inhibited mammalian cell line. J Cell Physiol 66: 325–333. 5884360 10.1002/jcp.1030660310

[69] Koth LL, Cambier CJ, Ellwanger A, Solon M, Hou L, et al (2010) DAP12 is required for macrophage recruitment to the lung in response to cigarette smoke and chemotaxis toward CCL2. J Immunol 184: 6522–6528 10.4049/jimmunol.0901171 20421649 PMC4091814

[70] Gold ES, Ramsey SA, Sartain MJ, Selinummi J, Podolsky I, et al (2012) ATF3 protects against atherosclerosis by suppressing 25-hydroxycholesterol-induced lipid body formation. J Exp Med 209: 807–817 10.1084/jem.20111202 22473958 PMC3328364

[71] Ramsey SA, Gold ES, Aderem A (2010) A systems biology approach to understanding atherosclerosis. EMBO Mol Med 2: 79–89 10.1002/emmm.201000063 20201031 PMC2992755

[72] Brasier AR (2010) The nuclear factor-kappaB-interleukin-6 signalling pathway mediating vascular inflammation. Cardiovasc Res 86: 211–218 10.1093/cvr/cvq076 20202975 PMC2912657

[73] Mallat Z, Besnard S, Duriez M, Deleuze V, Emmanuel F, et al (1999) Protective role of interleukin-10 in atherosclerosis. Circ Res 85: e17–e24. 10521249 10.1161/01.res.85.8.e17

[74] Virmani R, Burke AP, Farb A, Kolodgie FD (2002) Pathology of the unstable plaque. Prog Cardiovasc Dis 44: 349–356. 12024333 10.1053/pcad.2002.122475

[75] Kolodgie FD, Narula J, Burke AP, Haider N, Farb A, et al (2000) Localization of apoptotic macrophages at the site of plaque rupture in sudden coronary death. Am J Pathol 157: 1259–1268 10.1016/S0002-9440(10)64641-X 11021830 PMC1850160

[76] Laufer EM, Winkens MHM, Narula J, Hofstra L (2009) Molecular imaging of macrophage cell death for the assessment of plaque vulnerability. Arterioscler Thromb Vasc Biol 29: 1031–1038 10.1161/ATVBAHA.108.165522 19461053

[77] Li AC, Binder CJ, Gutierrez A, Brown KK, Plotkin CR, et al (2004) Differential inhibition of macrophage foam-cell formation and atherosclerosis in mice by PPARalpha, beta/delta, and gamma. J Clin Invest 114: 1564–1576 10.1172/JCI18730 15578089 PMC529277

[78] Thorp E, Kuriakose G, Shah YM, Gonzalez FJ, Tabas I (2007) Pioglitazone Increases Macrophage apoptosis and plaque necrosis in advanced atherosclerotic lesions of nondiabetic low-density lipoprotein receptor null mice. Circulation 116: 2182–2190 10.1161/CIRCULATIONAHA.107.698852 17967777

[79] Nakaya H, Summers BD, Nicholson AC, Gotto AM, Hajjar DP, et al (2009) Atherosclerosis in *LDLR*-knockout mice is inhibited, but not reversed, by the PPARgamma ligand pioglitazone. Am J Pathol 174: 2007–2014 10.2353/ajpath.2009.080611 19435790 PMC2684166

[80] Gong K, Zhou F, Huang H, Gong Y, Zhang L (2012) Suppression of GSK3β by ERK mediates lipopolysaccharide induced cell migration in macrophage through β-catenin signaling. Protein Cell 3: 762–768 10.1007/s13238-012-2058-x 22983902 PMC4875346

[81] van Gils JM, Derby MC, Fernandes LR, Ramkhelawon B, Ray TD, et al (2012) The neuroimmune guidance cue netrin-1 promotes atherosclerosis by inhibiting the emigration of macrophages from plaques. Nat Immunol 13: 136–143 10.1038/ni.2205 22231519 PMC3262880

[82] Sarzani R, Salvi F, Bordicchia M, Guerra F, Battistoni I, et al (2011) Carotid artery atherosclerosis in hypertensive patients with a functional LDL receptor-related protein 6 gene variant. Nutr Metab Cardiovasc Dis 21: 150–156 10.1016/j.numecd.2009.08.004 19833493

[83] Neumann J, Schaale K, Farhat K, Endermann T, Ulmer AJ, et al (2010) Frizzled1 is a marker of inflammatory macrophages, and its ligand Wnt3a is involved in reprogramming *Mycobacterium tuberculosis*-infected macrophages. FASEB J 24: 4599–4612 10.1096/fj.10-160994 20667980

[84] Marinou K, Christodoulides C, Antoniades C, Koutsilieris M (2012) Wnt signaling in cardiovascular physiology. Trends Endocrinol Metab 23: 628–636 10.1016/j.tem.2012.06.001 22902904

[85] Terrand J, Bruban V, Zhou L, Gong W, Asmar El Z, et al (2009) LRP1 controls intracellular cholesterol storage and fatty acid synthesis through modulation of Wnt signaling. J Biochem 284: 381–388 10.1074/jbc.M806538200 PMC2610522 18990694

[86] Go G-W, Srivastava R, Hernandez-Ono A, Gang G, Smith SB, et al (2014) The combined hyperlipidemia caused by impaired Wnt-LRP6 signaling is reversed by Wnt3a rescue. Cell Metab 19: 209–220 10.1016/j.cmet.2013.11.023 24506864 PMC3920193

[87] Fujino T, Asaba H, Kang M-J, Ikeda Y, Sone H, et al (2003) Low-density lipoprotein receptor-related protein 5 (LRP5) is essential for normal cholesterol metabolism and glucose-induced insulin secretion. Proc Natl Acad Sci USA 100: 229–234 10.1073/pnas.0133792100 12509515 PMC140935

[88] Hock MB, Kralli A (2009) Transcriptional control of mitochondrial biogenesis and function. Annu Rev Physiol 71: 177–203 10.1146/annurev.physiol.010908.163119 19575678

[89] Ishii T, Itoh K, Ruiz E, Leake DS, Unoki H, et al (2004) Role of Nrf2 in the regulation of CD36 and stress protein expression in murine macrophages: activation by oxidatively modified LDL and 4-hydroxynonenal. Circ Res 94: 609–616 10.1161/01.RES.0000119171.44657.45 14752028

[90] Puig O, Yuan J, Stepaniants S, Zieba R, Zycband E, et al (2011) A gene expression signature that classifies human atherosclerotic plaque by relative inflammation status. Circ Cardiovasc Genet 4: 595–604 10.1161/CIRCGENETICS.111.960773 22010137

[91] Hsieh P-C, Chiang M-L, Chang J-C, Yan Y-T, Wang F-F, et al (2012) DDA3 stabilizes microtubules and suppresses neurite formation. J Cell Sci 125: 3402–3411 10.1242/jcs.099150 22467851

[92] Zhang L, Shao H, Zhu T, Xia P, Wang Z, et al (2013) DDA3 associates with microtubule plus ends and orchestrates microtubule dynamics and directional cell migration. Sci Rep 3: 1681 10.1038/srep01681 23652583 PMC3647168

[93] Hsieh P-C, Chang J-C, Sun W-T, Hsieh S-C, Wang M-C, et al (2007) p53 downstream target DDA3 is a novel microtubule-associated protein that interacts with end-binding protein EB3 and activates beta-catenin pathway. Oncogene 26: 4928–4940 10.1038/sj.onc.1210304 17310996

[94] Nissen SE, Tuzcu EM, Schoenhagen P, Brown BG, Ganz P, et al (2004) Effect of intensive compared with moderate lipid-lowering therapy on progression of coronary atherosclerosis: a randomized controlled trial. JAMA 291: 1071–1080 10.1001/jama.291.9.1071 14996776

[95] Zhao XQ, Brown BG, Hillger L, Sacco D, Bisson B, et al (1993) Effects of intensive lipid-lowering therapy on the coronary arteries of asymptomatic subjects with elevated apolipoprotein B. Circulation 88: 2744–2753 10.1161/01.CIR.88.6.2744 8252687

[96] Ridker PM, Danielson E, Fonseca FAH, Genest J, Gotto AM, et al (2008) Rosuvastatin to prevent vascular events in men and women with elevated C-reactive protein. N Engl J Med 359: 2195–2207 10.1056/NEJMoa0807646 18997196

[97] Nissen SE, Nicholls SJ, Sipahi I, Libby P, Raichlen JS, et al (2006) Effect of very high-intensity statin therapy on regression of coronary atherosclerosis: the ASTEROID trial. JAMA 295: 1556–1565 10.1001/jama.295.13.jpc60002 16533939

[98] Parathath S, Grauer L, Huang L-S, Sanson M, Distel E, et al (2011) Diabetes adversely affects macrophages during atherosclerotic plaque regression in mice. Diabetes 60: 1759–1769 10.2337/db10-0778 21562077 PMC3114401

[99] Pfaffl MW (2001) A new mathematical model for relative quantification in real-time RT-PCR. Nucleic Acids Res 29: e45. 11328886 10.1093/nar/29.9.e45 PMC55695

[100] Cho HJ, Shashkin P, Gleissner CA, Dunson D, Jain N, et al (2007) Induction of dendritic cell-like phenotype in macrophages during foam cell formation. Physiol Genomics 29: 149–160 10.1152/physiolgenomics.00051.2006 17244792

[101] Hägg DA, Jernas M, Wiklund O, Thelle DS, Fagerberg B, et al (2008) Expression profiling of macrophages from subjects with atherosclerosis to identify novel susceptibility genes. Int J Mol Med 21: 697–704. 18506362

[102] Bolstad BM (2004) Low-level analysis of high-density oligonucleotide array data: background, normalization, and summarization. Ph.D. Thesis, Berkeley: University of California, Berkeley.

[103] Benjamini Y, Hochberg Y (1995) Controlling the false discovery rate: a practical and powerful approach to multiple testing. J Roy Stat Soc B 57: 289–300.

[104] Dai M, Wang P, Boyd AD, Kostov G, Athey B, et al (2005) Evolving gene/transcript definitions significantly alter the interpretation of GeneChip data. Nucleic Acids Res 33: e175 10.1093/nar/gni179 16284200 PMC1283542

[105] Affymetrix (2006) Identifying and validating alternative splicing events. Affymetrix Technical Note

[106] Okoniewski MJ, Miller CJ (2008) Comprehensive analysis of Affymetrix exon arrays using BioConductor. PLoS Comput Biol 4: e6 10.1371/journal.pcbi.0040006 18463711 PMC2323405

[107] Smyth GK (2004) Linear models and empirical Bayes methods for assessing differential expression in microarray experiments. Stat Appl Genet Mol Biol 3: Article3 10.2202/1544-6115.1027 16646809

[108] Dennis GJ, Sherman BT, Hosack DA, Yang J, Gao W, et al (2003) DAVID: database for annotation, visualization, and integrated discovery. Genome Biol 4: P3. 12734009 PMC193660

[109] Subramanian A, Tamayo P, Mootha VK, Mukherjee S, Ebert BL, et al (2005) Gene set enrichment analysis: a knowledge-based approach for interpreting genome-wide expression profiles. Proc Natl Acad Sci U S A 102: 15545–15550 10.1073/pnas.0506580102 16199517 PMC1239896

[110] Litvak V, Ratushny AV, Lampano AE, Schmitz F, Huang AC, et al (2012) A FOXO3-IRF7 gene regulatory circuit limits inflammatory sequelae of antiviral responses. Nature 10.1038/nature11428 PMC3556990 22982991

[111] Affymetrix (2005) Guide to probe logarithmic intensity error (PLIER) estimation. Affymetrix Technical Note

[112] Affymetrix (2005) Exon array background correction. First ed. Affymetrix Whitepaper.

[113] Dabney A, Storey JD (2003) QVALUE: the manual version 1.0. Computer Software User Guide, University of Washington, Department of Biostatistics

[114] Breiman L (2001) Random forests. Machine learning 45: 5–32.

[115] Siepel A, Bejerano G, Pedersen JS, Hinrichs AS, Hou M, et al (2005) Evolutionarily conserved elements in vertebrate, insect, worm, and yeast genomes. Genome Res 15: 1034–1050 10.1101/gr.3715005 16024819 PMC1182216

[116] Wu H, Caffo B, Jaffee HA, Irizarry RA, Feinberg AP (2010) Redefining CpG islands using hidden Markov models. Biostatistics 11: 499–514 10.1093/biostatistics/kxq005 20212320 PMC2883304

[117] Wingender E, Chen X, Hehl R, Karas H, Liebich I, et al (2000) TRANSFAC: an integrated system for gene expression regulation. Nucleic Acids Res 28: 316–319. 10592259 10.1093/nar/28.1.316 PMC102445

[118] Portales-Casamar E, Thongjuea S, Kwon AT, Arenillas D, Zhao X, et al (2010) JASPAR 2010: the greatly expanded open-access database of transcription factor binding profiles. Nucleic Acids Res 38: D105–D110 10.1093/nar/gkp950 19906716 PMC2808906

[119] Frith MC, Fu Y, Yu L, Chen J-F, Hansen U, et al (2004) Detection of functional DNA motifs via statistical over-representation. Nucleic Acids Res 32: 1372–1381 10.1093/nar/gkh299 14988425 PMC390287

[120] Nishida K, Frith MC, Nakai K (2009) Pseudocounts for transcription factor binding sites. Nucleic Acids Research 37: 939–944 10.1093/nar/gkn1019 19106141 PMC2647310

[121] Shannon P, Markiel A, Ozier O, Baliga NS, Wang JT, et al (2003) Cytoscape: a software environment for integrated models of biomolecular interaction networks. Genome Res 13: 2498–2504 10.1101/gr.1239303 14597658 PMC403769

[122] Wu X, Watson M (2009) CORNA: testing gene lists for regulation by microRNAs. Bioinformatics 25: 832–833 10.1093/bioinformatics/btp059 19181683 PMC2654799

[123] Smedley D, Haider S, Ballester B, Holland R, London D, et al (2009) BioMart–biological queries made easy. BMC Genomics 10: 22 10.1186/1471-2164-10-22 19144180 PMC2649164

[124] Reczko M, Maragkakis M, Alexiou P, Grosse I, Hatzigeorgiou AG (2012) Functional microRNA targets in protein coding sequences. Bioinformatics 28: 771–776 10.1093/bioinformatics/bts043 22285563

[125] Remm M, Storm CE, Sonnhammer EL (2001) Automatic clustering of orthologs and in-paralogs from pairwise species comparisons. J Mol Biol 314: 1041–1052 10.1006/jmbi.2000.5197 11743721

[126] Johnson AD, Handsaker RE, Pulit SL, Nizzari MM, O'Donnell CJ, et al (2008) SNAP: a web-based tool for identification and annotation of proxy SNPs using HapMap. Bioinformatics 24: 2938–2939 10.1093/bioinformatics/btn564 18974171 PMC2720775

