# Supplementary figures and images for: Epigenome-Guided Analysis of the Transcriptome of Plaque Macrophages during Atherosclerosis Regression Reveals Activation of the Wnt Signaling Pathway
Source: PLoS Genet. 2014 Dec 4;10(12):e1004828. doi: 10.1371/journal.pgen.1004828 (PMC4256277; doi:10.1371/journal.pgen.1004828)

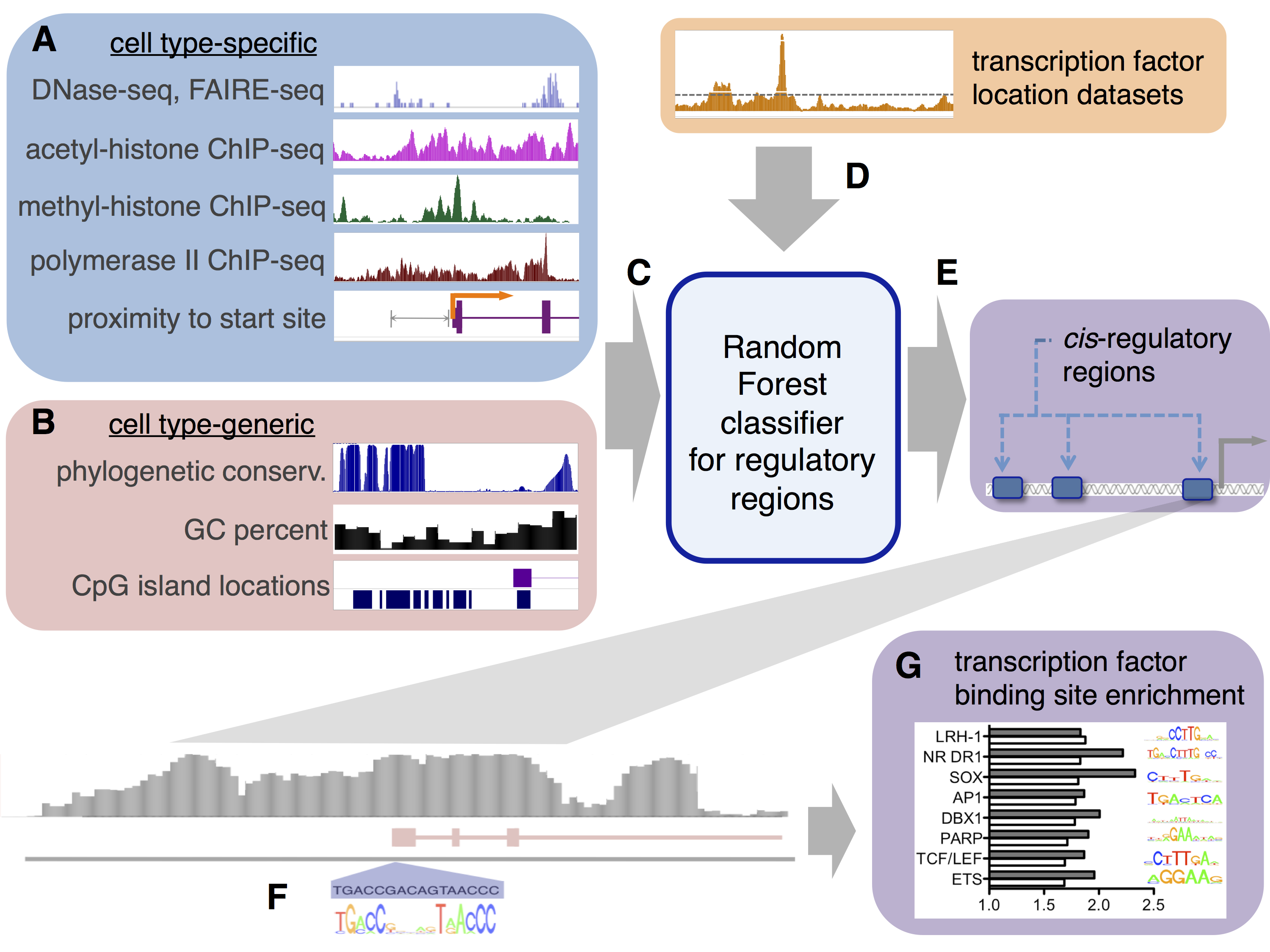

Supplement: Figure S1 — Diagram of REMINISCE method for detecting enrichments of transcription factor binding sites for specific transcription factors, within predicted regulatory regions. Cell type-specific epigenomic and chromatin information (A) are combined with cell type-generic genomic information (B) within an ensemble decision tree classifier, Random Forest (C). The classifier is trained to use the input features (A,B) to predict the locations of cis-regulatory regions, identified by combining transcription factor location datasets (D) from the specific cell type (macrophages). The classifier predicts cis-regulatory regions (E) upstream of genes that are differentially expressed in plaque macrophages in response to lipid lowering in vivo, and these regions are scanned to identify matches for transcription factor binding site motifs from a precompiled library of vertebrate motifs (F). For each transcription factor, frequencies per bp of predicted cis-regulatory region sequence for its binding sites are tabulated and tested for enrichment (G) above a background frequency from cis-regulatory regions for a list of genes that are expressed above background level in murine macrophages. See Methods section for details. (TIFF) [file pgen.1004828.s001.tiff]

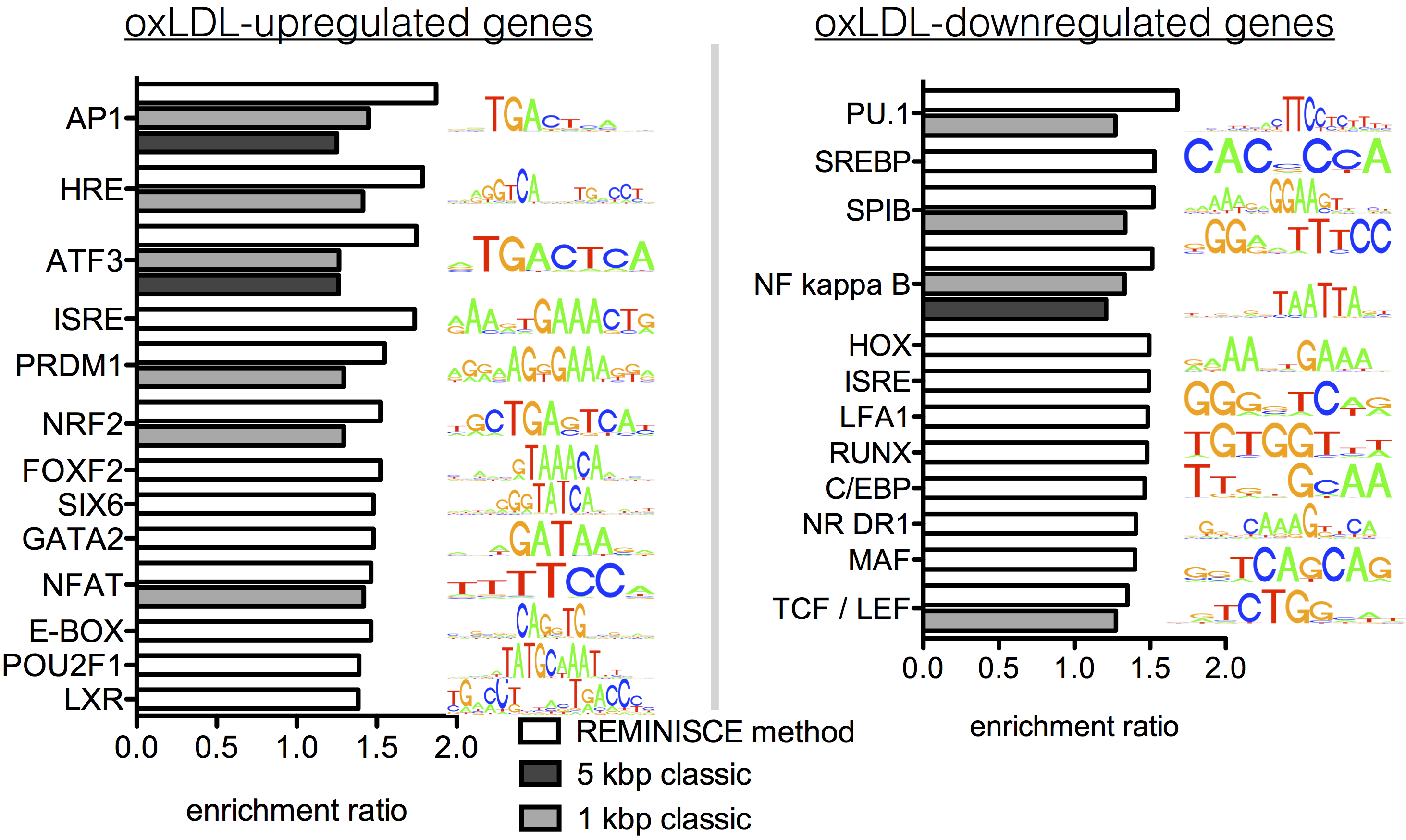

Supplement: Figure S2 — Sensitivity for in silico detection of enrichments of transcription factor binding sites is compared between the epigenome-guided method, REMINISCE, and approaches in which all noncoding sequence within ±1 kbp or ±5 kbp of transcription start sites are analyzed. The three analysis methods were applied to a transcriptome profiling study of macrophage foam cells in which mouse resident peritoneal macrophages (N = 3 independent replicate experiments; see Methods) were cultured with oxLDL or vehicle for 24 h. High-confidence sets of differentially expressed genes were identified (false discovery rate cutoff of 0.05 and minimum absolute fold-change of 2.0) as upregulated (94 genes) or downregulated (342 genes), and a background set of 2,000 genes that were randomly selected from the set of genes that were detected above background in at least one sample group (vehicle or oxLDL). The 5′ regulatory regions for the genes were scanned for matches to transcription factor binding site motifs using the epigenome-guided method, REMINISCE, as described in the Methods section (“REMINISCE method”, white bars) and by analyzing all noncoding sequence within ±1 kbp of transcription start sites (“1 kbp classic”, gray bars) or within ±5 kbp of transcription start sites (“5 kbp classic”, charcoal bars). For all three methods, the number of matches for a binding site motif in the biological gene sets (genes up- or downregulated by oxLDL) per kbp of sequence analyzed, was compared to the number that would be expected by chance for genes that are expressed above background in resident peritoneal macrophages. Bar group labels represent transcription factor binding site motifs. Bar lengths represent the ratio of the number of motif matches (per kbp of sequence analyzed) for the indicated oxLDL-response gene set (left, upregulated in oxLDL; right, downregulated in oxLDL) to the number of motif matches (per kbp of sequence analyzed) for the background set of genes. For each bar group (motif), a [file pgen.1004828.s002.tiff]

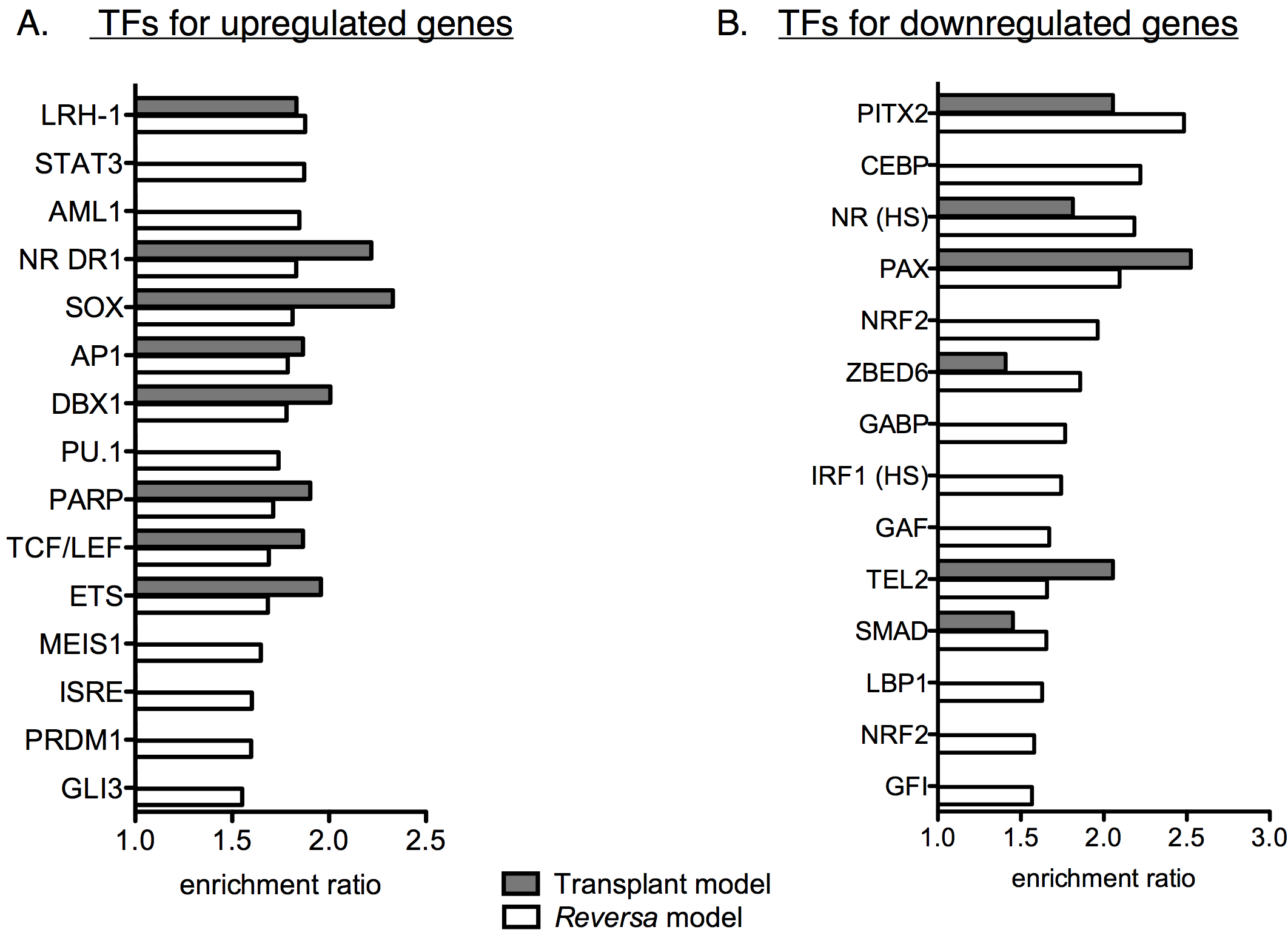

Supplement: Figure S3 — Motif scanning enrichment analysis for promoters of genes that are differentially expressed in plaque macrophages during plaque regression, in the Reversa and/or in the transplant regression models. Bars represent the ratio of the number of transcription factor (TF) binding site motif matches (per kbp of cis-regulatory sequence analyzed) for the indicated differentially expressed gene set, to the number of binding site motif matches per kbp for randomly generated sets of genes that are expressed above background in plaque CD68+ cells. Bar shading indicates the regression model from which the gene set was derived (white, Reversa model, gray, transplant regression model). A missing gray bar indicates that binding sites for the indicated TF binding site motif were not detected as significantly higher than the number expected by chance, for the transplant regression model. TF motifs shown on this figure were selected based on a significance threshold (see Methods) for enrichment for gene sets derived from the Reversa mouse, and thus, every TF shown here has a white bar. (A) TF binding site motifs for genes that are upregulated in CD68+ cells from regressing animals vs. control animals. (B) TF binding site motifs for genes that are downregulated in CD68+ cells from regressing animals vs. control animals. (TIFF) [file pgen.1004828.s003.tiff]

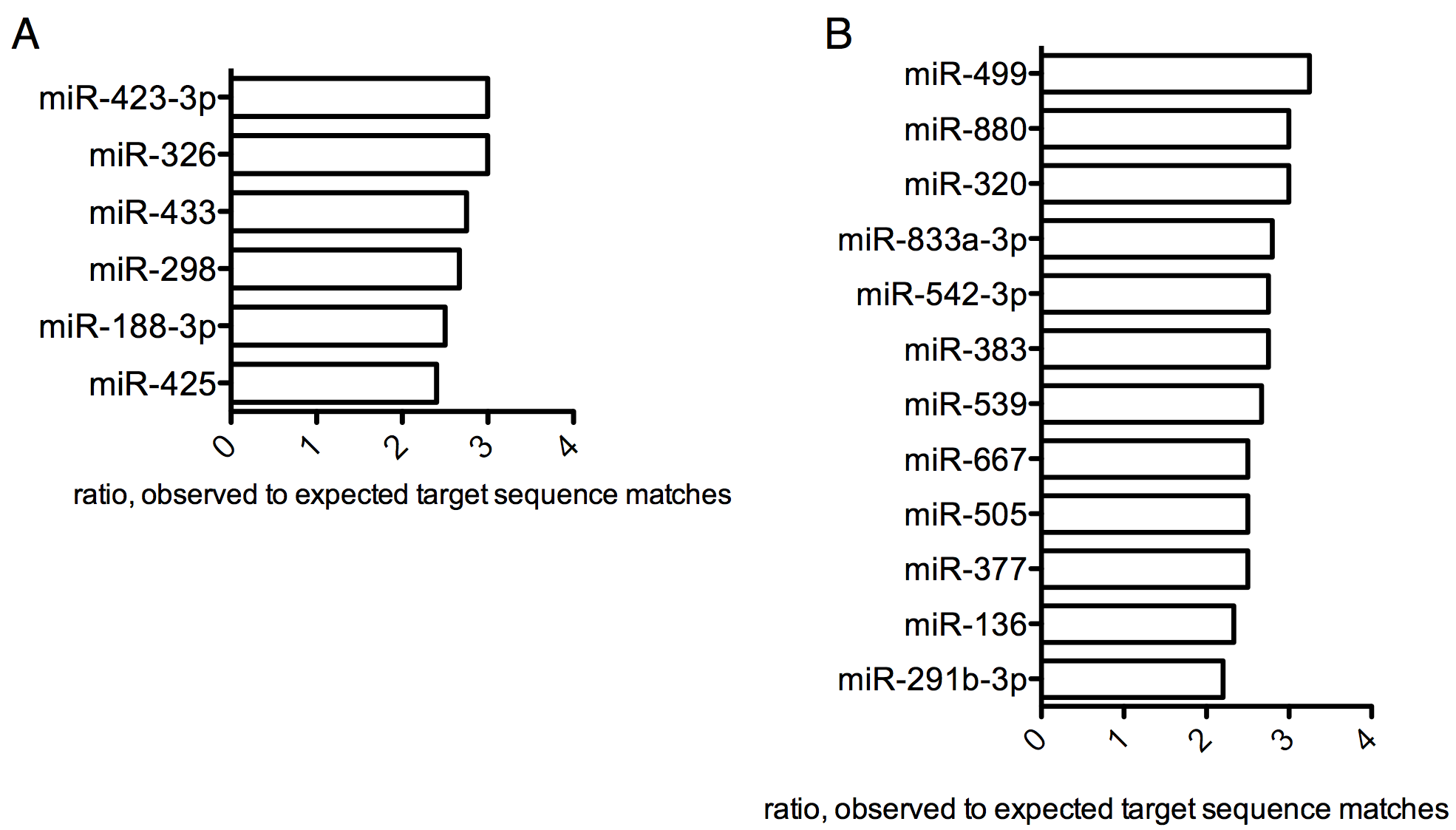

Supplement: Figure S4 — microRNA target sequence enrichment analysis identifies many microRNAs whose target sequences are overrepresented within the 3′ UTRs of genes that are differentially expressed in CD68+ cells in plaques in Mttp-inactivated vs. vehicle-treated Reversa mice. (A) Enrichment analysis for genes that are upregulated in Mttp-inactivated vs. vehicle-treated macrophages. Bars indicate the ratio of the number of target sequence matches within the gene set, vs. the number expected by chance for randomly selected sets of genes (see Methods). (B) Enrichment analysis for genes that are downregulated in Mttp-inactivated vs. vehicle-treated macrophages. (TIFF) [file pgen.1004828.s004.tiff]

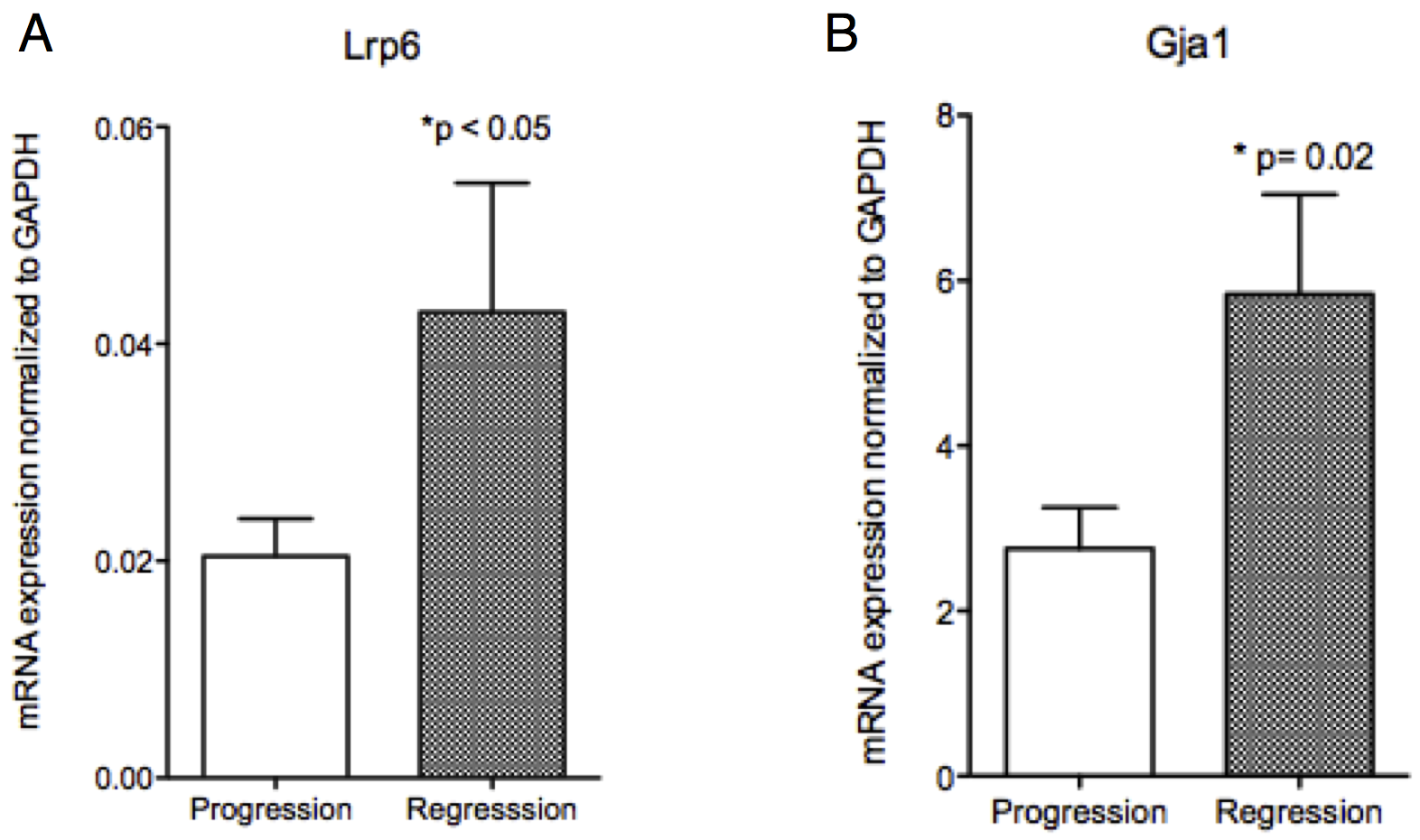

Supplement: Figure S5 — Upregulation of Wnt pathway target genes in CD68+ cells in regressing vs. progressing plaques. qPCR-measured relative mRNA levels for Wnt signaling pathway target genes Lrp6 (A) and Gja1 (B) in CD68+ cells in aortic grafts in Apoe −/− and WT recipient animals (N = 6 per genotype group) in the aortic transplant regression model on day five post-transplant show increased expression during plaque regression vs. progression. Bars, mean ± SE. P values reported are for an unpaired, two-tailed Student's t-test. (TIFF) [file pgen.1004828.s005.tiff]

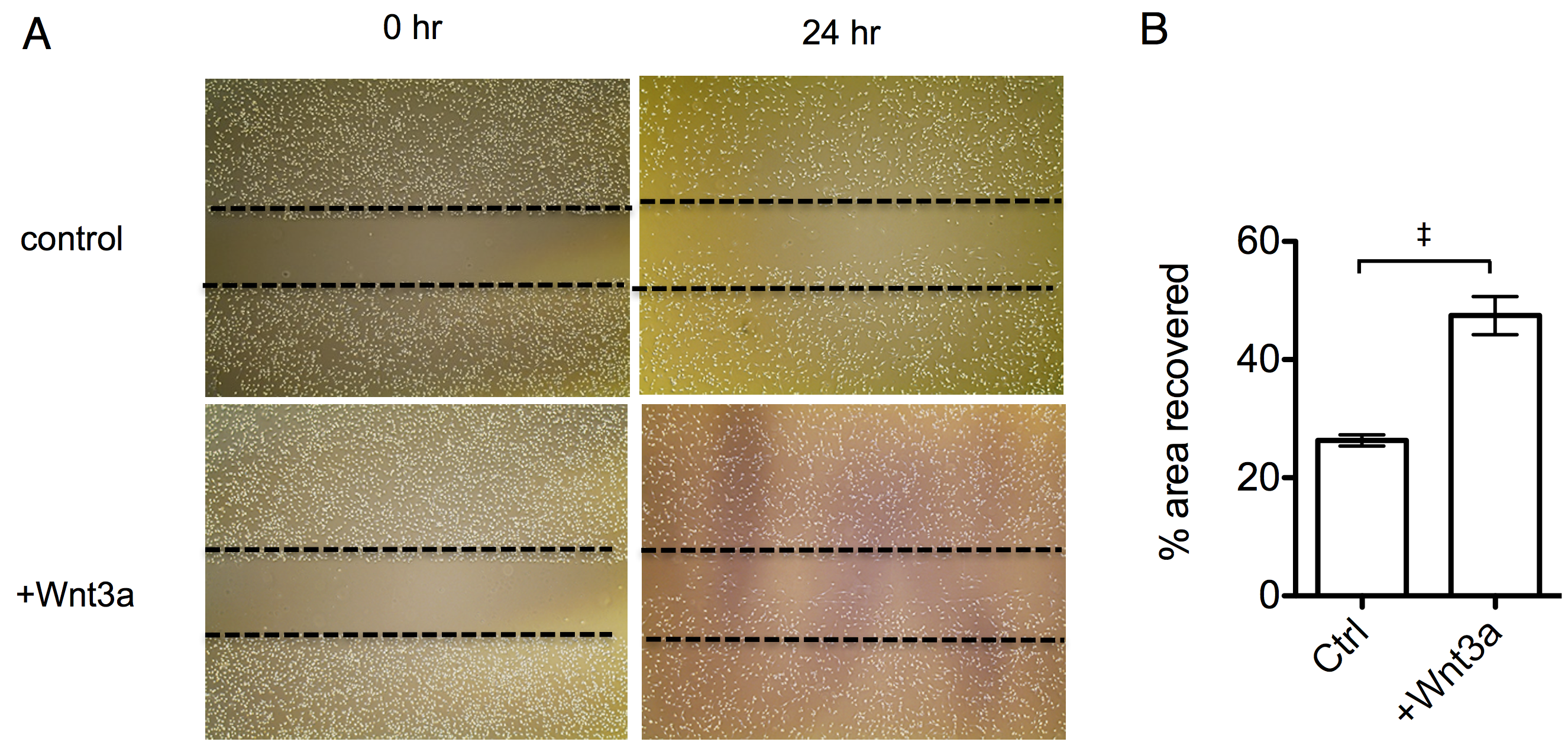

Supplement: Figure S6 — Canonical Wnt pathway activation stimulates macrophage migration in vitro. (A) Micrographs of a scratch-wound migration assay for primary murine macrophages incubated for 24 h in medium alone or medium plus Wnt3a (400 ng/mL). Dotted black lines demarcate the scratch region. (B) Quantification of percentage of scratch area recovered after 24 h incubation. Bars, mean ± SE (N = 4 replicates per sample group). (‡) P<0.01 (unpaired, two-tailed Student's t-test vs. control). (TIFF) [file pgen.1004828.s006.tiff]

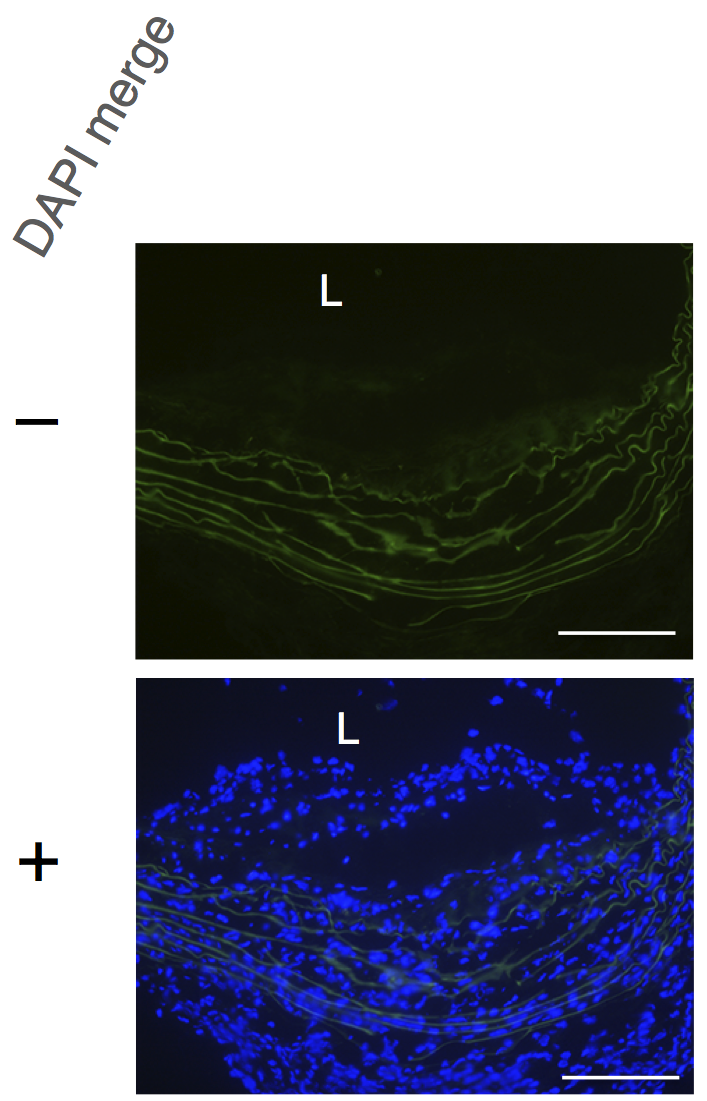

Supplement: Figure S7 — β-catenin immunofluorescence has minimal background signal in the arterial intima of aortic graft sections from the aortic transplant model of plaque regression. Representative images of the negative control (primary antibody excluded) are shown, with and without DAPI merge. The elastic lamina is identified by its autofluorescence. Green, β-catenin immunofluorescence; blue, DAPI fluorescence. Scale bar, 100 µm. (TIFF) [file pgen.1004828.s007.tiff]

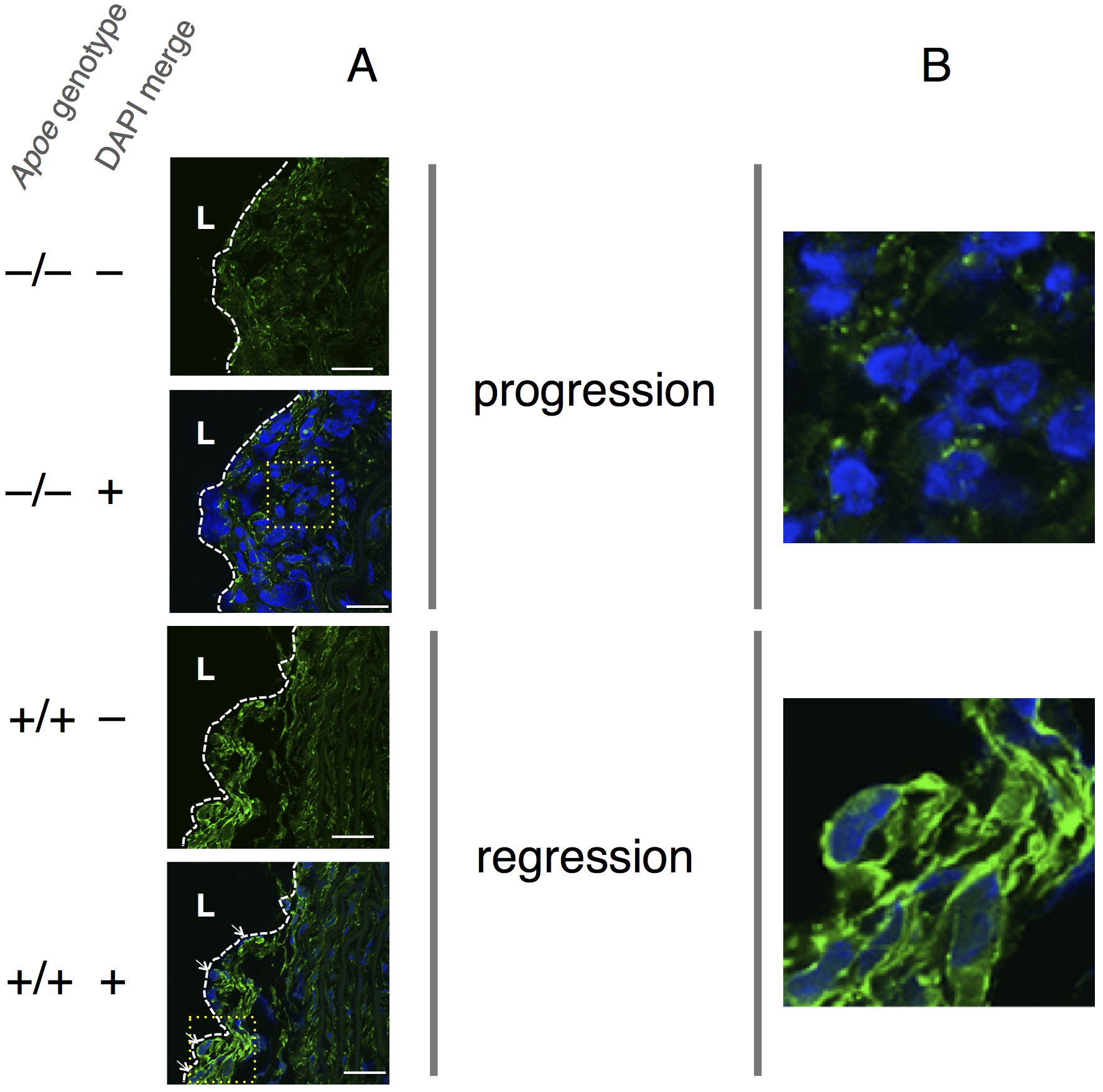

Supplement: Figure S8 — β-catenin immunofluorescence shows increased abundance in the nuclear area, in WT vs. Apoe−/− recipient mice in the aortic transplant regression model. (A) Images are confocal fluorescence micrographs from representative sections from a total of six grafts (N = 3 for each recipient animal genotype; six sections per graft). Green, β-catenin immunofluorescence; blue, DAPI fluorescence; dashed white line divides lumen from intima; scale bars, 20 µm. White arrows indicate areas of overlap of β-catenin and DAPI fluorescence signals in the regressing plaque section (bottom image). (B) Zoomed images of regions that are indicated with yellow dotted lines in the merged images from progressing and regressing plaques (A, second and fourth images from the top). (TIFF) [file pgen.1004828.s008.tiff]
